# Cell-free and alkylated hemoproteins improve survival in mouse models of carbon monoxide poisoning

**DOI:** 10.1172/jci.insight.153296

**Published:** 2022-11-08

**Authors:** Qinzi Xu, Jason J. Rose, Xiukai Chen, Ling Wang, Anthony W. DeMartino, Matthew R. Dent, Sagarika Tiwari, Kaitlin Bocian, Xueyin N. Huang, Qin Tong, Charles F. McTiernan, Lanping Guo, Elmira Alipour, Trevor C. Jones, K. Burak Ucer, Daniel B. Kim-Shapiro, Jesús Tejero, Mark T. Gladwin

**Affiliations:** 1 Heart, Lung, Blood and Vascular Medicine Institute,; 2 Division of Pulmonary, Allergy and Critical Care Medicine, and; 3 Department of Bioengineering, Swanson School of Engineering, University of Pittsburgh, Pittsburgh, Pennsylvania, USA.; 4 Department of Medicine, University of Maryland School of Medicine, Baltimore, Maryland, USA.; 5 Department of Orthopedics & Rehabilitation, University of Iowa, Iowa City, Iowa, USA.; 6 Department of Physics and; 7 Translational Science Center, Wake Forest University, Winston-Salem, North Carolina, USA.; 8 Department of Pharmacology and Chemical Biology, University of Pittsburgh, Pittsburgh, Pennsylvania, USA.

**Keywords:** Pulmonology, Therapeutics, Respiration, Toxicology

## Abstract

I.v. administration of a high-affinity carbon monoxide–binding (CO-binding) molecule, recombinant neuroglobin, can improve survival in CO poisoning mouse models. The current study aims to discover how biochemical variables of the scavenger determine the CO removal from the RBCs by evaluating 3 readily available hemoproteins, 2,3-diphosphoglycerate stripped human hemoglobin (StHb); *N*-ethylmaleimide modified hemoglobin (NEMHb); and equine myoglobin (Mb). These molecules efficiently sequester CO from hemoglobin in erythrocytes in vitro. A kinetic model was developed to predict the CO binding efficacy for hemoproteins, based on their measured in vitro oxygen and CO binding affinities, suggesting that the therapeutic efficacy of hemoproteins for CO poisoning relates to a high M value, which is the binding affinity for CO relative to oxygen (*K*_A,CO_/*K*_A,O2_). In a lethal CO poisoning mouse model, StHb, NEMHb, and Mb improved survival by 100%, 100%, and 60%, respectively, compared with saline controls and were well tolerated in 48-hour toxicology assessments. In conclusion, both StHb and NEMHb have high CO binding affinities and M values, and they scavenge CO efficiently in vitro and in vivo, highlighting their therapeutic potential for point-of-care antidotal therapy of CO poisoning.

## Introduction

Carbon monoxide (CO) is the most common non–drug-related human poisoning, accounting for tens of thousands of emergency department visits every year in the United States alone (1). CO exhibits a high affinity for ferrous heme, and CO binding impairs the function of critical hemoproteins, including hemoglobin and cytochrome *c* oxidase. In the case of hemoglobin, CO binding has 2 main detrimental effects: (a) CO binds tightly to the heme iron, decreasing the number of oxygen-binding sites available and thereby decreasing oxygen binding capacity, and (b) the CO-bound hemoglobin (carboxyhemoglobin [COHb]) allosterically stabilizes the high-affinity R-state tetramer, increasing the affinity for hemoglobin-bound oxygen and, therefore, decreasing oxygen release in the tissues. At high CO concentrations, CO also binds to the heme a3 group of cytochrome *c* oxidase, arresting electron transfer and aerobic metabolism (1, 2). The only current treatment for CO poisoning is inhalation of 100% oxygen under normobaric and/or hyperbaric (1.5–3 atmosphere) pressures. While these treatments increase the clearance rate of COHb from the blood, the efficacy of hyperbaric oxygen inhalation therapy is debated, largely due to delays in the initiation of hyperbaric treatment associated with patient transport (3). With current therapies, short-term mortality varies between 1 and 3% (4, 5). Up to 40% of survivors of CO poisoning suffer long-term neurocognitive sequelae (6, 7). Survivors have nearly double the long-term mortality of non–CO-poisoned patients (8). There remains a need for new point-of-care antidotal treatments for CO poisoning that can be rapidly deployed in the field and in emergency departments.

Our group has developed a potentially novel, hemoprotein-based CO scavenging therapeutic using an engineered variant of human neuroglobin (Ngb-H64Q-CCC). This protein, which exhibits a CO affinity 500-fold higher than that of hemoglobin (9), rapidly scavenges CO from CO-saturated RBCs in vitro and significantly decreases the half-life of COHb in mice. In a severe CO-poisoning mouse model, treatment with Ngb-H64Q-CCC reversed CO-induced hypotension and improved survival to 87.5%, compared with less than 10% survival for control animals treated with just i.v. fluid resuscitation (9). Mice treated with Ngb-H64Q-CCC after severe CO poisoning also showed improved tissue respiration and cytochrome *c* oxidase activity compared with CO-poisoned mice treated with only saline (10). While this molecule has the highest CO binding affinity identified to date and is highly effective as a CO antidote, it is not thoroughly understood how the biochemical characteristics of a CO scavenging agent determines the CO removal from the RBCs. Other efforts using heme-based small molecules are ongoing, highlighting the need for alternative molecules (11, 12).

To extend our knowledge about the role of CO-scavenging hemoproteins in antidotal pharmacology, the impact of ligand binding parameters (i.e., CO and oxygen affinity equilibrium constants, *K*_A,CO_ and *K*_A,O2_) on efficacy must be determined. Ngb-H64Q-CCC shows high affinity for both CO and oxygen, but it shows a higher overall selectivity for CO. This selectivity parameter is represented numerically by the M value, or the ratio of ligand binding affinities, *K*_A,CO_/*K*_A,O2_. It is logical to consider that high CO affinity will be advantageous for a CO scavenger, but selectivity for CO over oxygen must also be considered under aerobic conditions in the bloodstream. The exact relationship between in vitro ligand affinity and selectivity and the CO-scavenging efficacy in vivo is not well understood. Thus, a primary goal of this study is to establish benchmark criteria for CO binding affinity and selectivity that confer CO-scavenging efficacy in vivo.

Herein, the in vitro ligand binding properties were investigated, as well as in vivo safety/tolerability and CO-scavenging efficacy of 3 readily available hemoproteins with different affinities toward CO. Two of these proteins are human hemoglobin derivatives that are easily isolated in gram-scale quantities from expired, leukoreduced human donor packed RBCs. These 2 human hemoglobin derivatives employ different allosteric strategies to stabilize R-state hemoglobin, which exhibits higher CO affinity than T-state hemoglobin (13). In one hemoglobin derivative, 2,3-diphosphoglycerate (2,3-DPG) and other endogenous allosteric effectors were removed (stripped hemoglobin [StHb]). This approach stabilizes hemoglobin in the higher-affinity R-state, as compared with hemoglobin found in RBCs, where binding to 2,3-DPG stabilizes the lower-affinity T-state and thereby enhances oxygen delivery in vivo (14, 15). In the second hemoglobin derivative, Cys93 in the StHb β-subunit was chemically modified using *N*-ethylmaleimide (NEM) to form NEM-modified hemoglobin (NEMHb). This chemical modification also results in allosteric stabilization of R-state hemoglobin and enhanced ligand binding affinity compared with StHb (16, 17). Equine myoglobin (Mb) was also investigated as a third CO scavenger; however, this hemoprotein exhibits a lower CO binding affinity and M-value compared with StHb and NEMHb. Thus, Mb is expected to be less effective in CO scavenging than the hemoglobin-based molecules.

## Results

### Ligand binding properties of StHb and NEMHb.

Despite NEMHb being extensively studied from a biochemical and physiological standpoint, there are no reports of kinetic parameters for CO binding and dissociation parameters for this hemoglobin derivative. CO binding rate constants (*k*_on,CO_) were determined for StHb and NEMHb by directly monitoring CO rebinding following laser flash photolysis (Supplemental Figure 1, A–D; supplemental material available online with this article; https://doi.org/10.1172/jci.insight.153296DS1), and CO dissociation rate constants (*k*_off,CO_) were measured by the nitric oxide (NO) displacement method (Supplemental Figure 1, E–H) following standard procedures at room temperature (9). Oxygen dissociation rate constants (*k*_off,O2_) were determined by CO displacement (Supplemental Figure 1, I–L) (18). The relative affinity for CO and oxygen (M value) were measured by the ratio of COHb to oxyhemoglobin equilibrated to mixture of CO and oxygen gas with known concentration (19). Oxygen binding rate constants (*k*_on,O2_) were calculated indirectly from M value and other measured kinetic parameters. Kinetic parameters and binding rate constants are summarized in Table 1. Laser flash photolysis experiments revealed a slightly greater CO association rate constant for NEMHb (8.3 ± 0.2 × 10^6^ M^–1^s^–1^) compared with StHb (5.1 ± 0.2 × 10^6^ M^–1^s^–1^). The dissociation rate constants were similar between NEMHb (*k*_off,CO_ = 2.1 ± 0.2 × 10^–2^ s^–1^) and StHb (*k*_off,CO_ = 1.3 ± 0.2 × 10^–2^ s^–1^), with NEMHb exhibiting a slightly larger dissociation rate constant (Table 1). Rate constants for oxygen dissociation for NEMHb (*k*_off,O2_ = 1.8 ± 0.3 × 10^–1^ s^–1^) and StHb (*k*_off,O2_ = 1.5 ± 0.3 × 10^–1^ s^–1^) were similar. Measured M value for StHb (242 ± 4) was greater than the measured M value for NEMHb (231 ± 3), as shown in Supplemental Figure 2. *k*_on,O2_ calculated for NEMHb (3.1 ± 0.3 × 10^7^ M^–1^s^–1^) and StHb (2.4 ± 0.3 × 10^7^ M^–1^s^–1^) were similar. These data show that NEMHb and StHb have very similar affinities for CO and are both similar to the reported CO affinity for R-state hemoglobin (Table 1).

### In vitro CO transfer from RBCs to extracellular hemoprotein scavengers.

**Figure 1:**
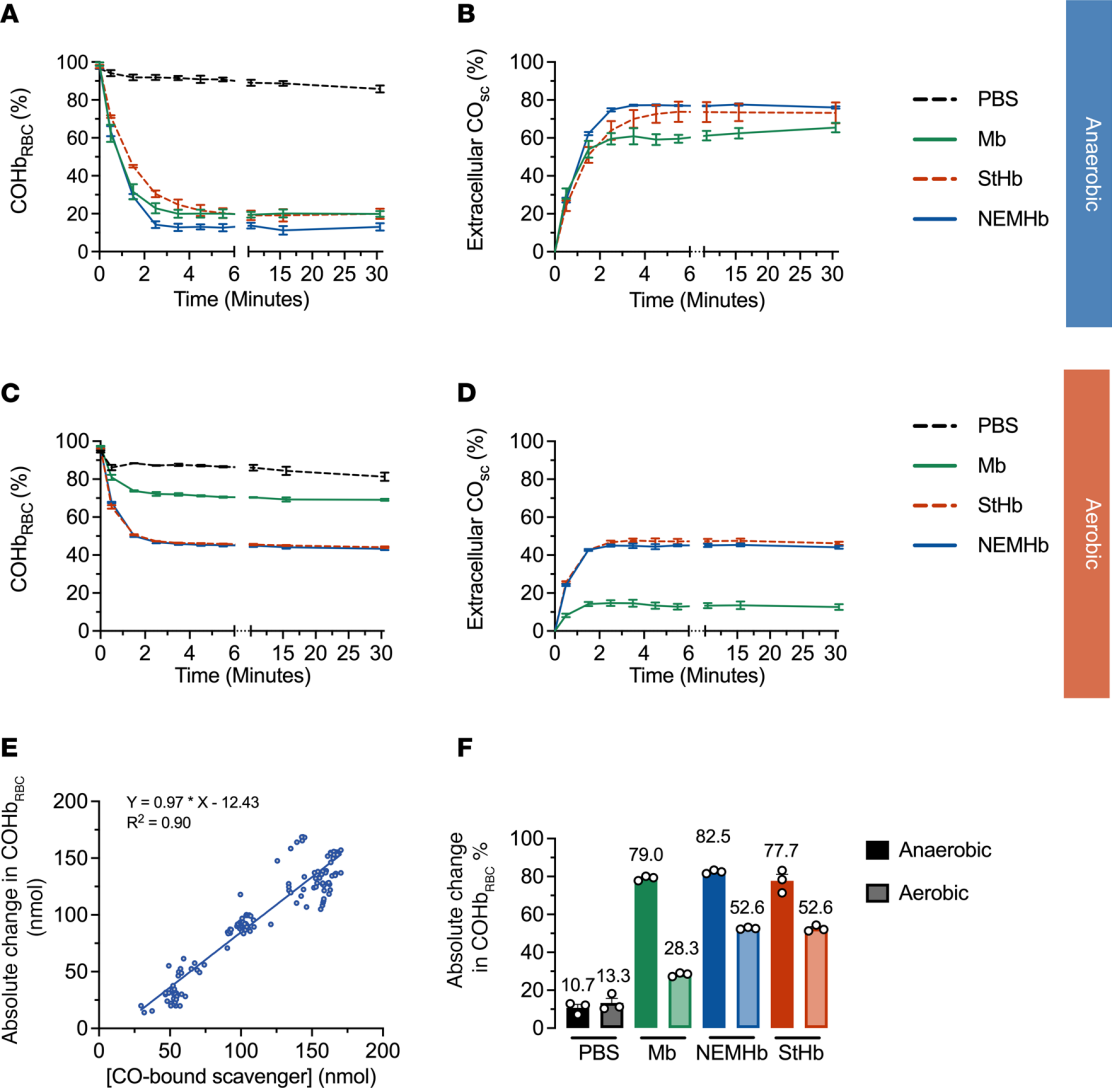
In vitro CO transfer from CO-saturated RBCs to extracellular ferrous scavengers under anaerobic and aerobic conditions. CO transfer kinetics observed after mixing equimolar (in heme) amounts of RBC-encapsulated carboxyhemoglobin and extracellular scavengers at room temperature (25°C). (**A**–**D**)Cells were incubated and mixed with PBS (black), Mb (green), NEMHb (blue), or StHb (red). Changes in the fractions of CO-bound hemoglobin in RBCs (COHb_RBC_) and CO-bound extracellular scavenger (CO_sc_) under anaerobic conditions (**A** and **B**) and under aerobic conditions (**C** and **D**). Data are shown as the mean ± SEM (*n* = 3). (**E**) Combined data comparing concentrations of CO-bound scavengers and CO removed from RBC-encapsulated COHb (absolute change in COHb_RBC_) for all scavengers under anaerobic and aerobic conditions. The solid blue line shows a simple linear regression fit to the data, and the associated linear equation is displayed. (**F**) Absolute change in COHb_RBC_% at equilibrium (after 30 minutes) under aerobic and anaerobic conditions. Displayed numbers are the mean values and error bars are SEM (*n* = 3).

To assess the ability of the hemoproteins to scavenge CO from RBC-encapsulated COHb, each hemoprotein was incubated with CO-saturated RBCs under aerobic and anaerobic conditions. Changes in CO-bound species were monitored using spectrophotometric techniques as previously reported (9). Mixing CO-saturated RBCs with equimolar (in heme) hemoproteins (StHb, NEMHb, and Mb) at room temperature (25°C) resulted in a rapid decline in fraction of COHb to total hemoglobin encapsulated in RBCs (COHb_RBC_) with a concomitant rise in fraction of CO-bound scavenger to total extracellular scavenger (CO_sc_; Figure 1, A–D). CO transfer was nearly complete after ~2 minutes for all proteins under both aerobic and anaerobic conditions, and CO_sc_ levels were stable, even after 30 minutes. A plot comparing the decrease in the concentration of RBC-encapsulated COHb and that of CO-bound scavenger yields a linear regression with a slope of 0.97, consistent with the expected 1:1 transfer of CO from the RBC to the extracellular scavengers (with minimal escape of CO from the solution via diffusion) under both anaerobic and aerobic conditions (Figure 1E). Hemolysis, which may artificially inflate the amount of extracellular COHb, does not appear to occur on the timescale of these experiments, as extracellular hemoglobin concentrations were unchanged upon removal of RBCs at the end of the experiment.

The extent of CO transfer from RBC-encapsulated COHb to each extracellular scavenger was greater under anaerobic conditions than under aerobic conditions, demonstrating that oxygen competition for the heme sites decreases the magnitude of CO scavenging. Figure 1F shows that, under anaerobic conditions, the change in the fraction of COHb in RBCs at equilibrium was approximately 80% for each extracellular scavenger compared with only 10.7% ± 1.8% for the PBS control. The presence of oxygen attenuated CO scavenging by extracellular hemoproteins. For example, absolute change in COHbRBC% for NEMHb decreased from 82.5% ± 0.5% under anaerobic conditions to only 52.6% ± 0.3% under aerobic conditions. Similarly, under anaerobic conditions, absolute change in COHb_RBC_% for StHb was 77.7% ± 3.4% under anaerobic conditions versus 52.6% ± 0.9% under aerobic conditions. The ability of Mb to scavenge CO from CO-saturated RBCs was greatly attenuated by the presence of oxygen, as absolute change in COHb_RBC_% decreased from 79.0% ± 0.7% under anaerobic conditions to only 28.3% ± 0.6% under aerobic conditions. This observation is consistent with lower CO selectivity over oxygen for Mb (M = 16) compared with StHb (M = 242) and NEMHb (M = 231) (Table 1).

### CO binding efficacy and therapeutic effects of hemoprotein scavengers in a severe CO-poisoning mouse model.

**Figure 2:**
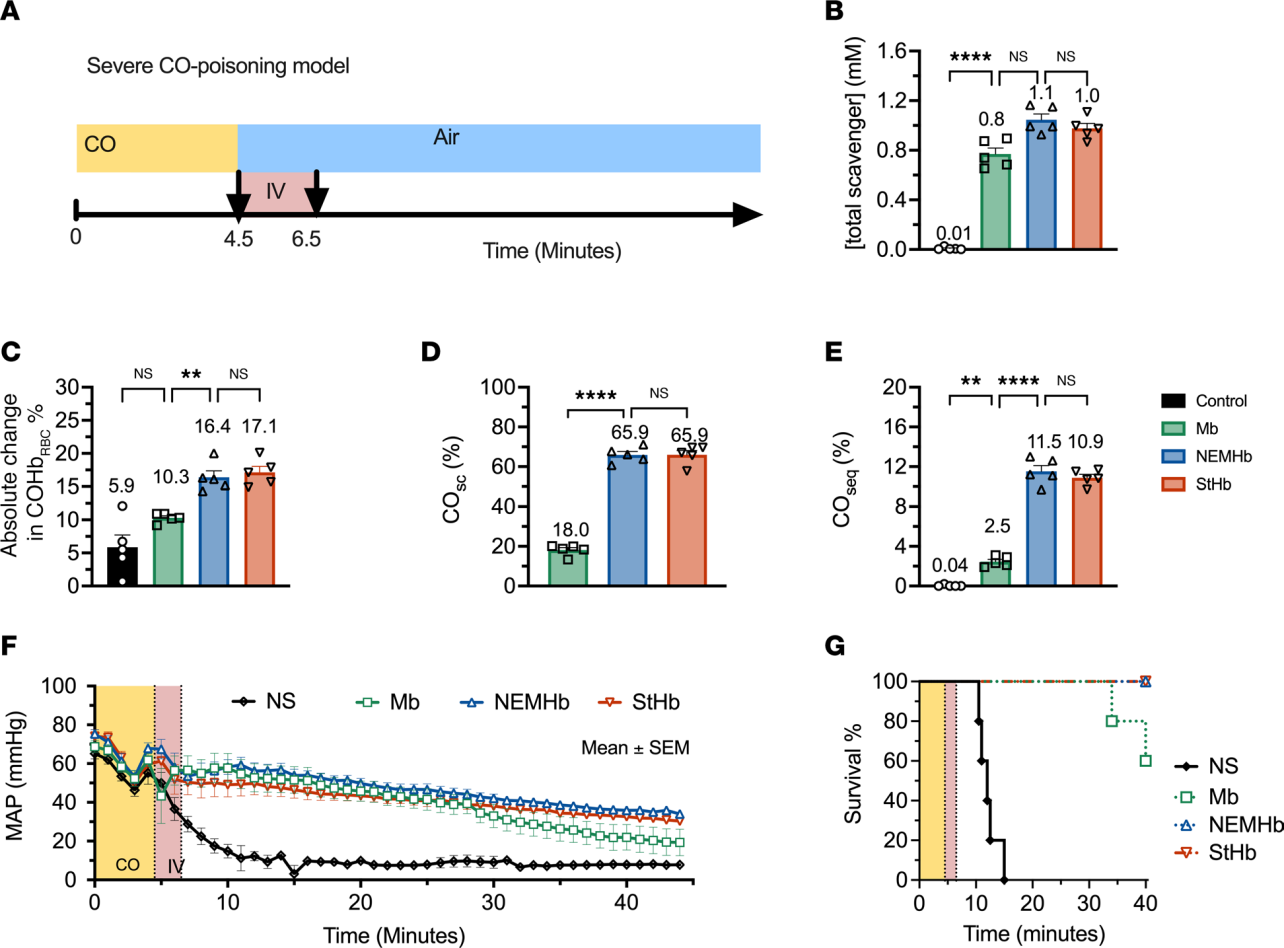
Comparison of ferrous hemoprotein intervention in severe–CO-poisoning mouse model. (**A**) Severe–CO-poisoning mouse model schematic. CO inhalation (30,000 ppm) lasts for 4.5 minutes. “CO” (yellow) indicates a period of CO inhalation, “Air” (blue) indicates a period of breathing air, and “IV” (red) indicates the 2-minute i.v. infusion of NS, Mb, NEMHb, or StHb. Blood samples were collected at the start and end of infusion. The arrow shows the time blood samples withdrawn. (**B**) Blood concentration of hemoprotein scavengers measured at the end of infusion. (**C**) Absolute change in COHb_RBC_% after infusion. (**D**) Percent of CO-bound exogenous hemoprotein at the end of infusion. (**E**) Percent of CO_seq_ at the end of infusion. (**F**) Changes in MAP as a function of time in CO exposure and hemoprotein treatment for all mice (survivors and nonsurvivors). Survivor-only MAP is shown in Supplemental Figure 3. Data are shown as mean ± SEM. (**G**) Kaplan-Meier survival curves for mice in the lethal–CO-poisoning model. Survival was 0% for the NS group 40 minutes after CO exposure, 60.0% for Mb, and 100.0% for StHb and NEMHb. Data are shown as ± SEM for each group (*n* = 5). *P* values were calculated for **D** and **E** by 1-way ANOVA with Tukey test to correct for multiple comparison. Log-rank (Mantel-Cox) test was used to compare the survival curves in **G**.

Infusion of NEMHb and StHb each significantly reduced the amount of RBC-encapsulated COHb present in the circulation of mice exposed to a lethal dose of CO in a model of severe CO-related asphyxiation with intubation and resuscitation (9). In this severe-poisoning model, anesthetized mice were ventilated and exposed to 3% CO (30,000 parts per million CO, premixed with 21% oxygen and 76% nitrogen) for 4.5 minutes, followed by immediate i.v. infusion of hemoprotein scavenger or normal saline (NS) control (Figure 2A). The COHb_RBC_ reached 96.9% ± 0.2 % at the end of the 4.5-minute CO inhalation period, resulting in 0% survival and a median survival time of 12.0 minutes in mice treated with NS (Figure 2G). Mice treated with hemoproteins were given the same molar dose of 100 μmol/kg body weight. To reach this dose, an adequate volume (10 mL/kg body weight, between 240 and 320 μL) of hemoprotein solution (10 mM in heme) was infused immediately at the end of the 4.5-minute period of CO inhalation. Arterial blood concentrations of NEMHb and StHb at the end of 2-minute infusion were 1.1 ± 0.1 mM and 1.0 ± 0.1 mM, respectively (Figure 2B and Supplemental Figure 6D). In comparison, blood concentrations of Mb were somewhat lower (0.8 ± 0.1 mM). This difference in blood concentration may be related to faster renal excretion or extravasation; however, no dedicated pharmacokinetics analysis was performed. Hematocrit, measured in a separate cohort of mice in the same model, decreased from 45.%2 ± 0.6% before CO inhalation and i.v. infusion to 35.3% ± 0.7% after infusion, reflecting the blood dilution by infused fluid. No significant differences in hematocrit were observed between treatment groups. In a separated 72-hour pharmacokinetics study, the half-life of NEMHb was 2.2 hours (Supplemental Figure 7A), and NEMHb was observed in the urine 30 minutes after injection (Supplemental Figure 7B).

The fraction of CO-bound hemoglobin in RBCs, determined from spectral deconvolution of lysed RBCs, decreased after infusion for NEMHb (absolute change in COHb_RBC_% = 16.4% ± 1.0%) and StHb (absolute change in COHb_RBC_% = 17.1% ± 0.9%), and the magnitudes of these changes were significantly greater than that of the NS control (absolute change in COHb_RBC_% = 5.9% ± 1.9%; Figure 2C and Supplemental Figure 6, A and B). At the same time point (immediately following infusion), significant fractions of CO-bound NEMHb (CO_sc_ = 65.9% ± 3.9%) and StHb (CO_sc_ = 65.9% ± 4.9%), determined from spectral deconvolutions of blood plasma, were observed (Figure 2D and Supplemental Figure 6C). The effect of supplemental 100% oxygen was tested in a separated cohort and was found to not significantly affect the absolute change in COHb_RBC_% of NS (5.9% ± 1.9% with air, 6.5% ± 0.6% with 100% oxygen; *P* = 0.981) nor NEMHb (16.4% ± 1.0% with air, 19.8% ± 1.2% with 100% oxygen; *P* = 0.278) (Supplemental Figure 4, A and B).

Several factors contribute to the observed absolute change in COHb_RBC_%, including CO elimination via lungs, redistribution of CO from blood to tissues, and sequestration by exogenous hemoprotein scavengers. Fraction of CO scavenged by hemoproteins to total initial CO was defined as CO sequestration (CO_seq_), which was not affected by CO elimination via lungs and redistribution to tissues. It was also used for comparison of CO-binding efficacy and was estimated by blood concentration of RBC-encapsulated hemoglobin, initial COHb_RBC_ percentage, hematocrit, and correction for blood dilution by infusions (Supplemental Table 1).

Values for CO_seq_ were estimated to be 11.5% ± 1.3% for NEMHb and 10.9% ± 0.8% for StHb (Figure 2E). In contrast to NEMHb and StHb, only 18.0% ± 2.8% CO-bound Mb was observed at the end of infusion (Figure 2D and Supplemental Table 1), along with an absolute change in COHb_RBC_% of only 10.3% ± 0.3% and an estimated CO_seq_ of 2.5% ± 0.5%. One-way ANOVA showed no significant difference in absolute change in COHb_RBC_% between Mb and the NS control group (*P* = 0.08; Figure 2E). These differences in CO binding efficacy between hemoglobin derivatives and Mb are consistent with the CO binding properties observed in vitro: NEMHb and StHb have ~27-fold higher CO binding affinities and ~14-fold higher CO selectivity over oxygen compared with Mb (Table 1). In a separate cohort, partial pressure of oxygen (PO_2_) kept decreasing after infusion in the NS group, while a recovery of PO_2_ was observed with StHb treatment (Supplemental Figure 5).

Therapeutic effects were also investigated in terms of mean arterial pressure (MAP), heart rate (HR), and survival for each treatment group. As shown previously (9, 10), all mice examined in the severe CO-poisoning model suffered hypotension induced by CO exposure (Figure 2F). I.v. infusion of NEMHb, StHb, or Mb at the same dose improved MAP (Figure 2F) and HR (Supplemental Figure 3B), and improvements were sustained for at least 40 minutes after initial exposure to CO. In contrast, blood pressures for animals in the control group collapsed within minutes of exposure. Survivor-only MAP is shown in Supplemental Figure 3A. The absolute change in COHbRBC percentage of NEMHb, StHb, and Mb significantly improved the survival rate from 0% for NS to 60.0% for Mb and 100.0% for StHb and NEMHb (Figure 2G).

### CO binding efficacy and therapeutic effects for StHb and NEMHb dose-response study in the severe–CO-poisoning model.

Dose-response studies for StHb and NEMHb were evaluated using the same severe–CO-poisoning model as for the hemoprotein comparison study. Mice were administered doses between 0 and 150 μmol/kg body weight for StHb or NEMHb (0–2,400 mg/kg) in a NS vehicle, and outcomes were compared with NS infusion alone. A volume of 10 mL/kg body weight was infused at an initial hemoprotein concentration ranging from 1 to 15 mM to achieve target dose levels. At least 5 mice were tested at each dosing level.

**Figure 3:**
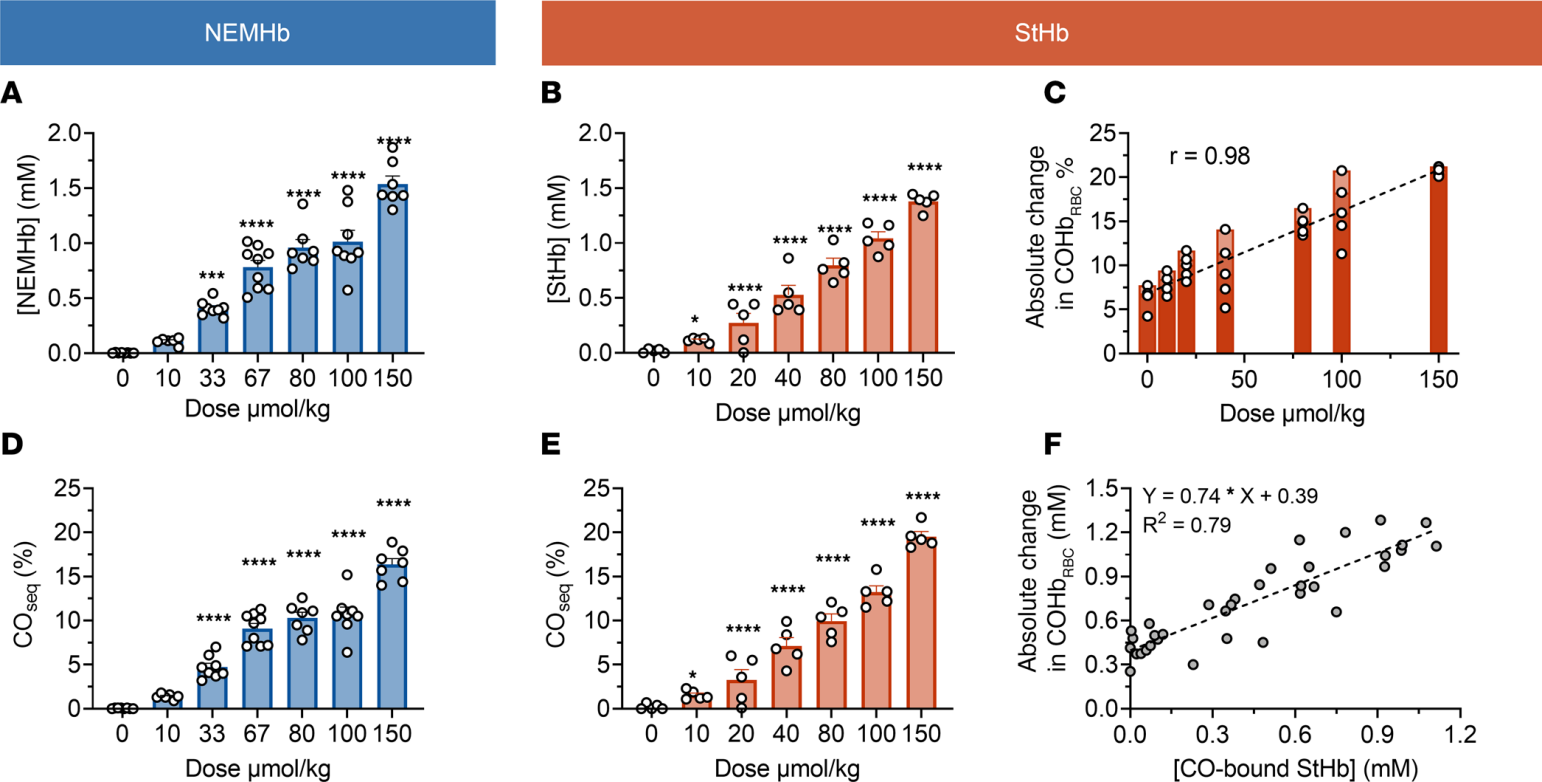
CO binding efficacy for dose-response studies of StHb and NEMHb in severe–CO-poisoning model. (**A** and **B**) Blood concentration at the end of infusion of NEMHb (**A**) and StHb (**B**) after CO exposure in the severe–CO-poisoning model. (**C**) Dose-dependent absolute change in COHb_RBC_% at the end of infusion of StHb. The dashed line represents a linear fit to the average value of absolute change in COHb_RBC_% for each dose group. (**D** and **E**) Estimated fraction of CO_seq_ at the end of infusion for NEMHb (**D**) and StHb (**E**). (**F**) Correlation between concentration of absolute change in COHb_RBC_% and CO-bound StHb. Data was shown as mean ± SEM (*n* = 5 for each group in BCEF, *n* =6–9 for each group in **A** and **D**). One-way ANOVA was used to determine the *P* values for **A**, **B**, **D**, and **E**, with Tukey test to correct for multiple comparison with the control groups.

Blood concentrations of extracellular NEMHb and StHb rose proportionally with dose (Figure 3, A and B). At the maximum dosage of 150 μmol/kg, the concentration of NEMHb (15 mM) and StHb (15 mM) approached 1.5± 0.1 mM and 1.4± 0.0 mM immediately after infusion, respectively. A decrease in COHb_RBC_ was observed in StHb and was found to be significantly correlated with dose (Spearman *r* = 0.98; Figure 3C). Values for absolute change in COHb_RBC_% were not available for the NEMHb dose-response study. Estimated fraction of CO_seq_ by the exogenous scavenger increased proportionally with dose (Figure 3, D and E). A linear relationship between decrease in RBC COHb and CO-bound StHb was observed (*R*^2^ = 0.79 for simple linear regression; Figure 3F), indicating the dose-dependent CO scavenging by StHb.

**Figure 4:**
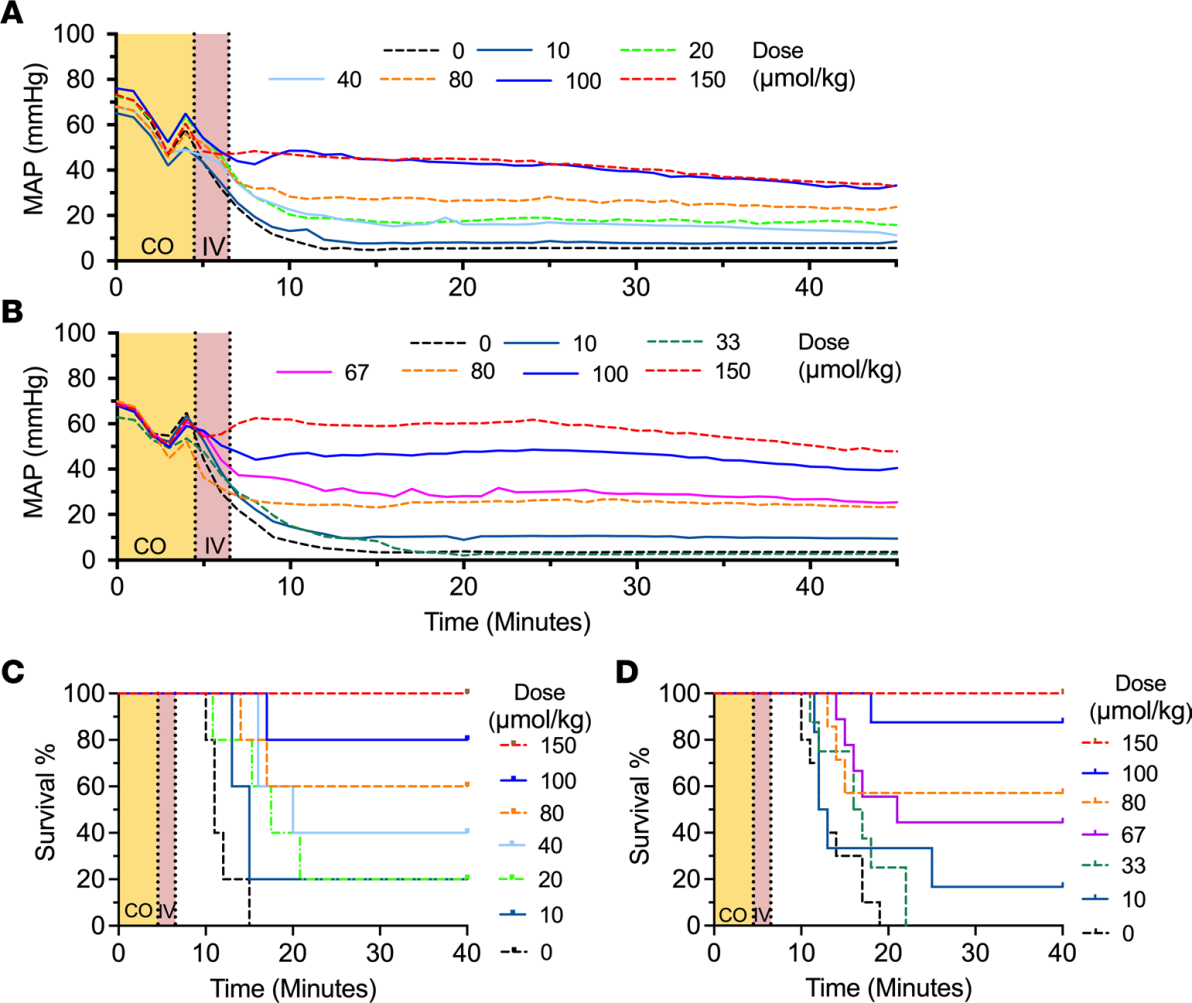
Therapeutic effects for dose-response studies of StHb and NEMHb in severe–CO-poisoning model. (**A** and **B**) Time course of MAP for StHb (**A**) and NEMHb (**B**) are expressed as means without errors for all mice (including survivors and nonsurvivors). Survivor-only MAP is shown in Supplemental Figure 3. (**C** and **D**) Kaplan-Meier survival curves for StHb (**C**; log-rank test, *P* < 0.0001) and NEMHb (**D**; log-rank test, *P* < 0.0001). “CO” (yellow) indicates the 4.5-minute CO inhalation, and “IV” (red) indicates the 2-minute infusion in the time course. (*n* =5 for each group in **A** and **C**, *n* =6–9 for each group in **B** and **D**).

Significant dose response effects on shock and mortality were observed. Profound hypotension, similar to that observed in the hemoprotein comparison study, was induced by CO inhalation and was reversed in a dose-dependent manner with both StHb and NEMHb infusions (Figure 4, A and B). Survivor-only MAP is shown in Supplemental Figure 3, C and D. Survival after infusions of StHb and NEMHb also significantly correlated with dose, as shown in Kaplan-Meier survival curves (Figure 4, C and D). Lower dose (40 μmol/kg) StHb resulted in an absolute change in COHbRBC percentage of 9.4% (Figure 3C) and increased survival rate to 40% compared with 0% of NS (Figure 4C). These results were consistent with Mb with an absolute change in COHb_RBC_ percentage of 10.3% (Figure 2C) and survival rate of 40% (Figure 2G).

### CO-binding efficacy for hemoprotein scavengers in moderate–CO-poisoning models.

**Figure 5:**
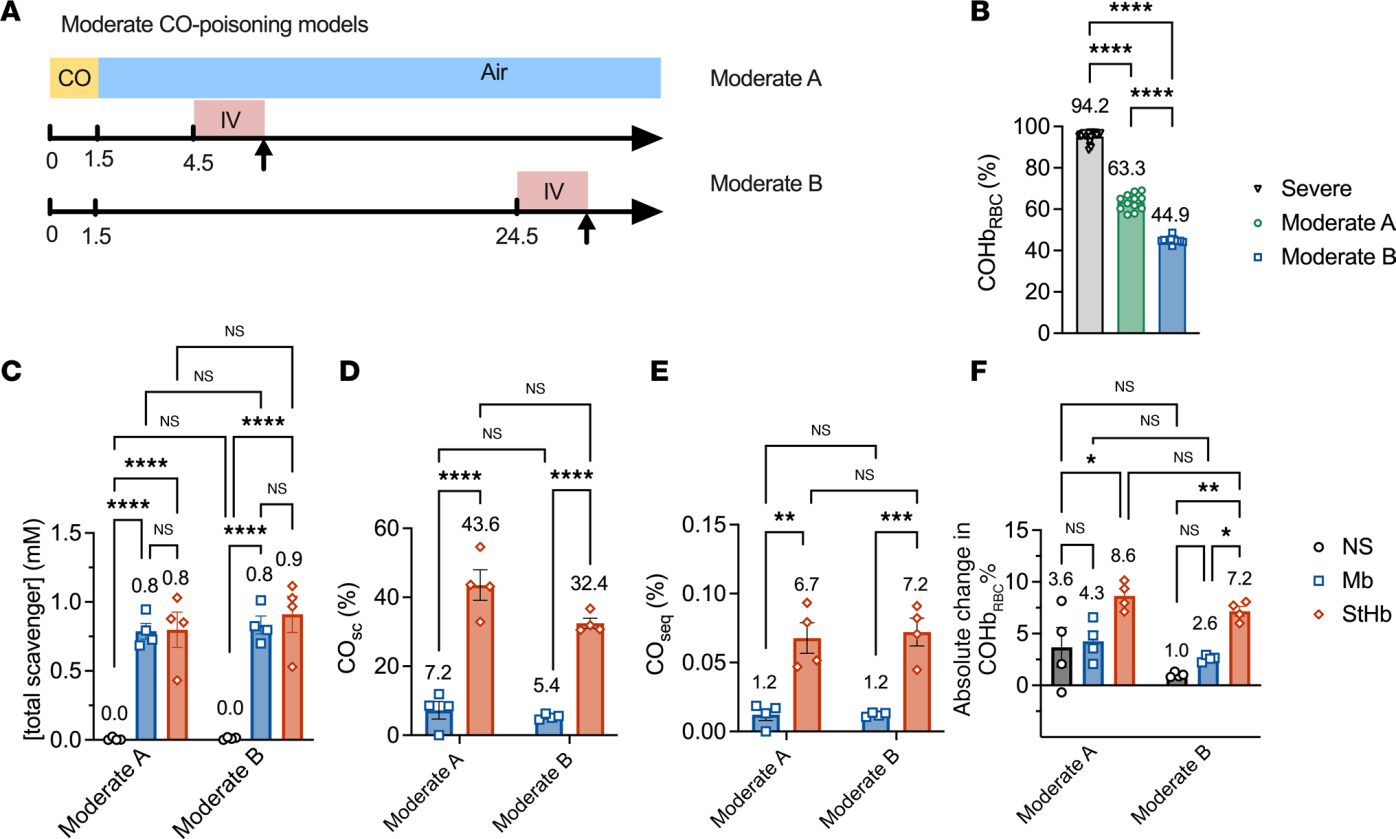
Efficacy of hemoprotein scavengers in moderate–CO-poisoning model. (**A**) The moderate–CO-poisoning mouse models schematic. Mice are anesthetized and mechanically ventilated throughout the course of the experiment. “CO” (yellow) indicates a period of CO inhalation of 1.5 minutes, “Air” (blue) indicates a period of breathing air, and “IV” (red) represents the 2-minute infusion. The arrow shows the time blood samples withdrawn at the start and end of infusion. For the Moderate model A, hemoprotein infusion starts 4.5 minutes after initial CO exposure, while for the Moderate model B, hemoprotein infusion starts 24.5 minutes after initial CO exposure. (**B**) Comparison of COHb_RBC_ prior to infusion for Moderate models A and B. (**C**) Blood concentrations of exogenous scavenger at the end of infusion. (**D**) CO_sc_ percentage at the end of infusion. (**E**) Fraction of CO_seq_ at the end of infusion. (**F**) Absolute change in COHb_RBC_ (%) after the 2-minute infusion. *P* values were calculated by 2-way ANOVA for **B**–**F**. Data represents mean ± SEM (*n* = 7 for each treatment group).

Two less severe, nonlethal CO-poisoning mouse models were developed (Figure 5A) to simulate 2 kinds of “real-world” situations for the treatment — e.g., a fast treatment with the antidote administered quickly after the exposure (a “first responders” treatment) versus a delayed treatment that would correspond to a later infusion (an “emergency room” situation). CO exposure time was shortened from 4.5 minutes to 1.5 minutes for both models. In Moderate model A, infusion occurred 4.5 minutes after initial CO exposure, while in Moderate model B, infusion occurred 24.5 minutes after initial CO exposure. As the latency period between CO exposure and infusion increased, the fraction of CO-bound hemoglobin decreased; COHb_RBC_ was 63.3% ± 1.1% at the start of infusion in model A and 44.9% ± 0.4% at the start of infusion in model B (Figure 5B). This observation is consistent with CO elimination from the circulation by redistribution to tissues and/or release via the lungs.

For these moderate–CO-poisoning studies, hemoproteins were infused at the same molar dose (100 μmol/kg), and hemoprotein concentration in blood was measured at the end of the 2-minute infusion period. There was no significant difference of hemoprotein scavenger concentrations in blood between the 2 moderate models. Measured hemoprotein blood concentrations immediately after infusion were 0.8 ± 0.6 mM in model A and 0.8 ± 0.1 mM in model B for Mb, and they were 0.8 ± 0.1 mM in model A and 0.9 ± 0.1 mM in model B for StHb (*P* > 0.9 by multiple comparison of 2-way ANOVA; Figure 5C).

StHb exhibited significantly greater efficacy than Mb (Figure 5, D and E). In both moderate models, the fraction of CO-bound Mb (7.2% ± 2.5%, model A; 5.4% ± 0.4%, model B) was significantly lower than that of StHb (43.6% ± 4.4%, model A, *P* < 0.001; 32.4% ± 1.5%, model B, *P* < 0.001; by 2-way ANOVA). Estimated fraction for CO_seq_ were significantly smaller for Mb (CO_seq_ = 1.2% ± 0.4 % in model A; 1.2% ± 0.1 % in model B) compared with StHb (CO_seq_ = 6.7% ± 1.1 % in model A, *P* = 0.0014; 7.2% ± 1.0 % in model B, *P* = 0.0007; by 2-way ANOVA). Interestingly, estimates of CO_seq_ for Mb and StHb do not vary as a function of initial COHb_RBC_ levels in the moderate–CO-poisoning models. Figure 5F shows that absolute change in COHb_RBC_% was higher for StHb (absolute change in COHb_RBC_% = 8.6% ± 0.7% in model A, *P* = 0.0002; 7.2% ± 0.5% in model B, *P* = 0.0024, by 2-way ANOVA) but not significantly different for Mb (absolute change in COHb_RBC_% = 4.3% ± 1.0% in model A, *P* = 0.0076; 2.6% ± 1.5% in model B, *P* = 0.8314, by 2-way ANOVA) compared with NS (absolute change in COHb_RBC_% = 3.6% ± 1.9% in model A; 1.0% ± 0.1% in model B; *P* < 0.0001, by 2-way ANOVA).

### Forty-eight–hour safety and tolerability assessment of NEMHb and StHb treatments.

**Figure 6:**
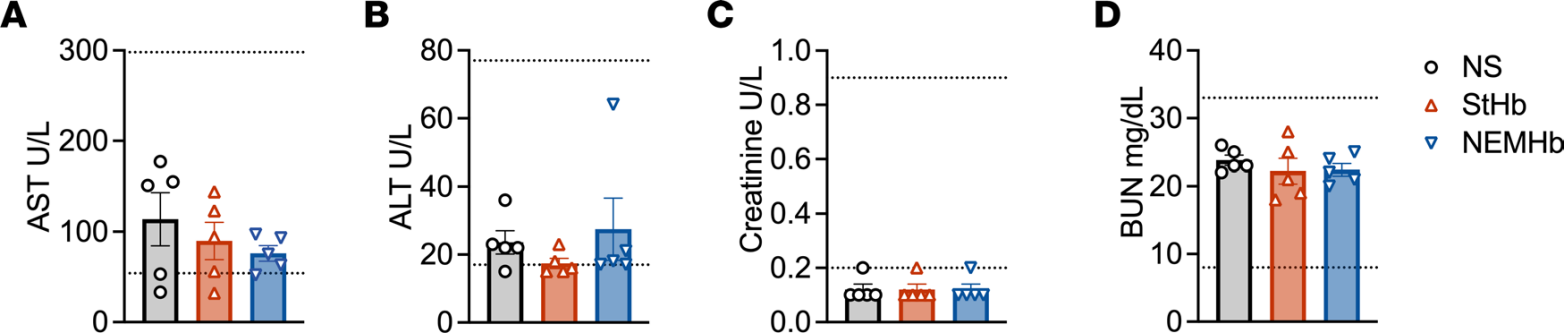
Assessment of liver and kidney function in mice treated with StHb or NEMHb. Mice were treated with StHb or NEMHb at a dose of 100 μmol/kg body weight, and outcomes were compared with a control group of mice given normal saline (NS) by i.v. infusion. (**A**–**D**) Blood samples were taken 48 hours after infusion followed by chemical analysis to quantify plasma concentrations of liver biomarkers, aspartate transaminase (AST) (**A**) and alanine transaminase (ALT) (**B**), as well as kidney biomarkers, creatinine (**C**) and blood urea nitrogen (BUN) (**D**). Dotted lines indicate upper and lower limits for healthy mice. Data are shown as mean ± SEM (*n* = 5 in each group). One-way ANOVA was used for comparison.

I.v. infusion of NEMHb and StHb at a therapeutic dose was well tolerated in a separate cohort of nonpoisoned, healthy animals. For safety studies, mice were infused with exogenous hemoprotein at a dose of 100 μmol/kg body weight via tail vein catheter and monitored for 48 hours after infusion. Outcomes for mice treated with NEMHb and StHb were compared with those of animals infused with NS, and there were 5 mice for each treatment group and the NS control group. All mice survived 48 hours after i.v. infusion, and no differences in behavior were observed between NEMHb and StHb treatment groups and the NS control group. No significant changes in biomarkers for liver function (alanine transaminase and aspartate transaminase; Figure 6, A and B) or kidney function (blood urea nitrogen and creatinine; Figure 6, C and D) were observed 48 hours after infusion. No difference was found between NS, StHb, and NEMHb for animal behavior, body weight, and complete blood count (Supplemental Table 2). No changes in clinical outcomes or clinical biomarker of organ function compared with NS control indicate safety and tolerability for i.v. injection of NEMHb and StHb at therapeutic doses.

### Development of a kinetic model to predict CO binding, clearance, and scavenging efficacy in vivo.

To further assess CO scavenging efficacy of exogenous hemoproteins, a predictive kinetic model was developed to estimate absolute change in COHb_RBC_% at the end of the 2-minute infusion for the severe–CO-poisoning mouse model. Using the biochemical modeling software Complex Pathway Simulator (COPASI, version 4.29) (20), CO transfer kinetics from RBC-encapsulated COHb (RCO) to exogenous CO scavenger molecules (X) were modeled by inputting kinetic parameters for Equations 1–4.


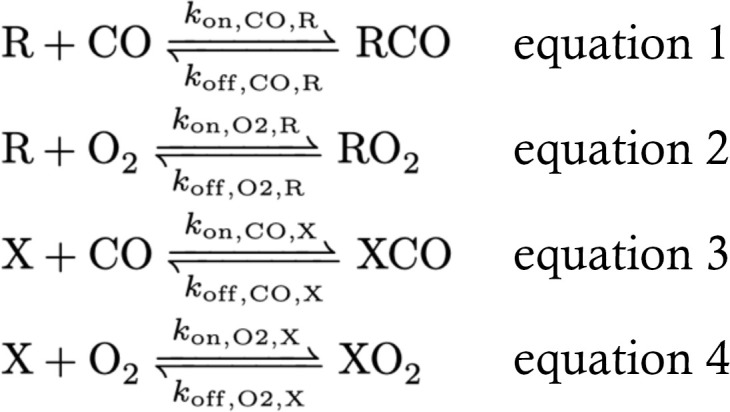


Where *k*_on_ and *k*_off_ are the binding and dissociation rate constants of the indicated ligand (oxygen or CO), the scavenger (X), and RBC hemoglobin (R). Additionally, irreversible loss of CO from the system (*k*_COloss_), either through diffusion at the liquid-air interface in vitro or through tissue distribution and exhalation in the circulatory system in vivo, is included in the model by Equation 5:


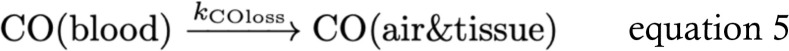


Kinetic rate constants for ligand binding (*k*_on_) and ligand dissociation (*k*_off_) for hemoglobin and extracellular CO scavengers were determined from in vitro experiments and used to compute ligand affinity values (Table 1). Given the presence of CO and oxygen in the system, the model assumes that all hemoglobin proteins (RBC-encapsulated hemoglobin and extracellular StHb and NEMHb) are in the R-state; therefore, R-state affinity parameters were used. *k*_COloss_ was determined from changes in absolute change in COHb_RBC_ for PBS control experiments in vitro and NS control groups from the hemoprotein comparison (Figure 2D) and StHb dose-response studies (Figure 3C) in vivo. Rate constants, initial protein, and ligand concentrations are summarized in Supplemental Table 3.

**Figure 7:**
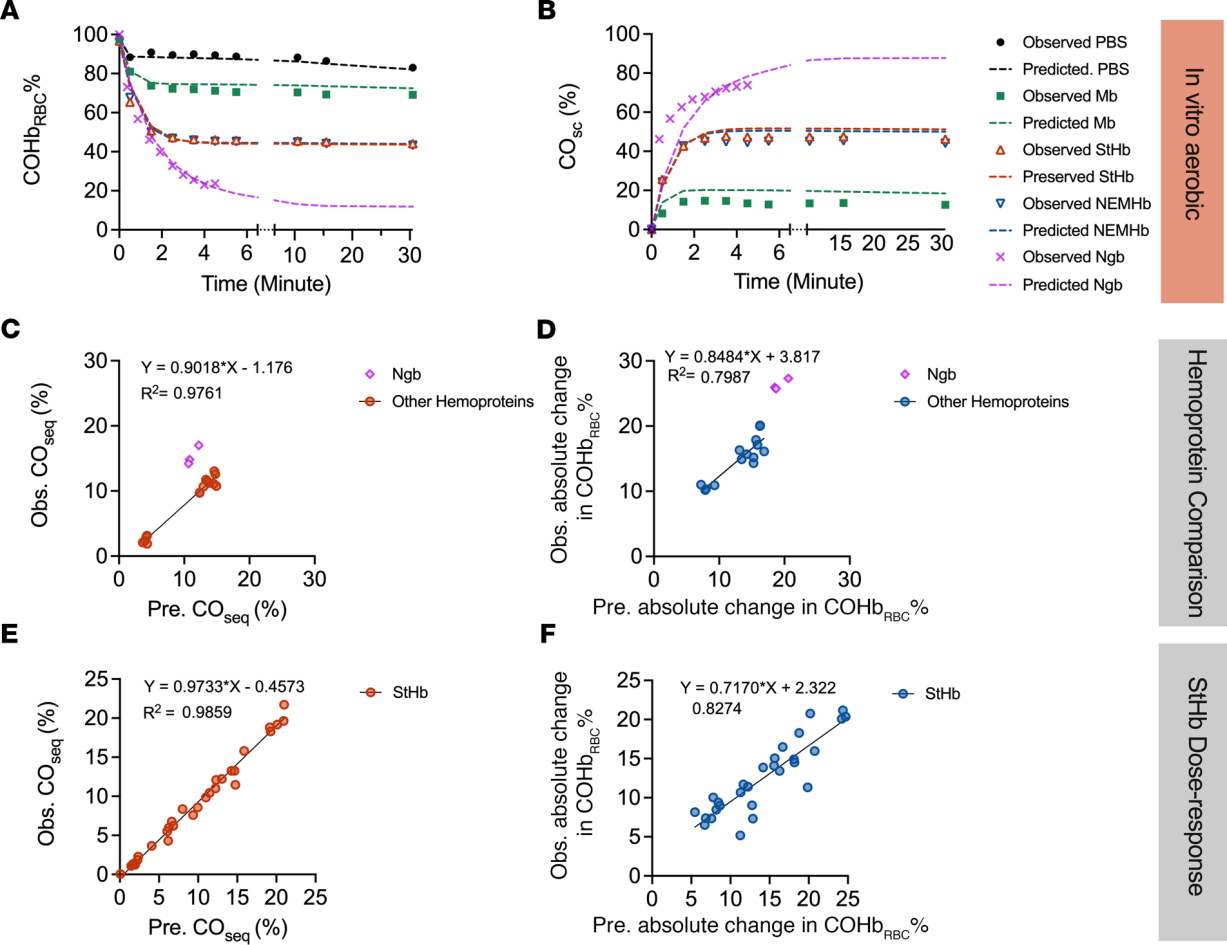
Correlation between predictions by kinetics modeling and observations. (**A** and **B**) Observed (Obs.) and Predicted (Pre.) time course of COHb_RBC_ (**A**) and extracellular CO_sc_ (**B**) for in vitro study under aerobic conditions at 25°C except Ngb-H64Q-CCC data. Observed Ngb-H64Q-CCC data came from published data (9) of in vitro CO experiments at 37°C. Predicted Ngb data are made based on experiments at 25°C. Symbols show the observed value; dashed lines show predicted values. (**C** and **D**) Comparison of observed and predicted CO_seq_ (**C**) and absolute change in COHb_RBC_% (**D**) for the hemoprotein comparison study in the severe–CO-poisoning mouse model. Observed Ngb-H64Q-CCC data were adopted from published data with the same mouse model (9). (**E** and **F**) Comparison of observed and predicted CO_seq_ (**E**) and absolute change in COHb_RBC_% (**F**) for the StHb dose-response study in the severe–CO-poisoning mouse model. The *R*^2^ is nonparametric Spearman correlation coefficient for each figure. Black solid lines show the simple linear regression.

Kinetic traces modeling the loss of CO from RBC-encapsulated COHb in vitro are in agreement with experimental data for samples mixed with StHb, NEMHb, Mb, and Ngb-H64Q-CCC (Figure 7A). Ngb-H64Q-CCC decreased COHb_RBC_ percentage much more than other hemoproteins. Predicted equilibrium values for the final CO_sc_ were slightly higher than those observed experimentally (Figure 7B).

The model was then validated with 2 data sets representing different typical situations. Predictive performance of the model is depicted visually for decrease in COHb_RBC_ and fraction of CO_seq_ by exogenous scavenger for each validation data set, respectively. The hemoprotein comparison study in the severe–CO-poisoning mouse model examined hemoproteins with different affinity for CO and oxygen at high initial COHb_RBC_ before treatment (Figure 7, C and D). The StHb dose-response study tested a range of molar doses (0–150 μmol/kg body weight; Figure 7, E and F).

Supplemental Table 4 summarizes the predictive performance of the kinetics model for the in vivo studies, including slope, Y-intercept, coefficient of determination (*R*^2^), and root mean squared error (RMSE). Although there is no published prediction of CO scavenging efficacy for direct comparison, the predictive performance is considered accurate with *R*^2^ of 0.80–0.99, a slope of 0.72–0.97, and a Y-intercept and RMSE less than half SD of the measured data. The intercept for CO_seq_ in the hemoprotein study was higher than other data sets, and this difference could possibly be related to lower blood concentration of Mb at the same molar dose.

**Figure 8:**
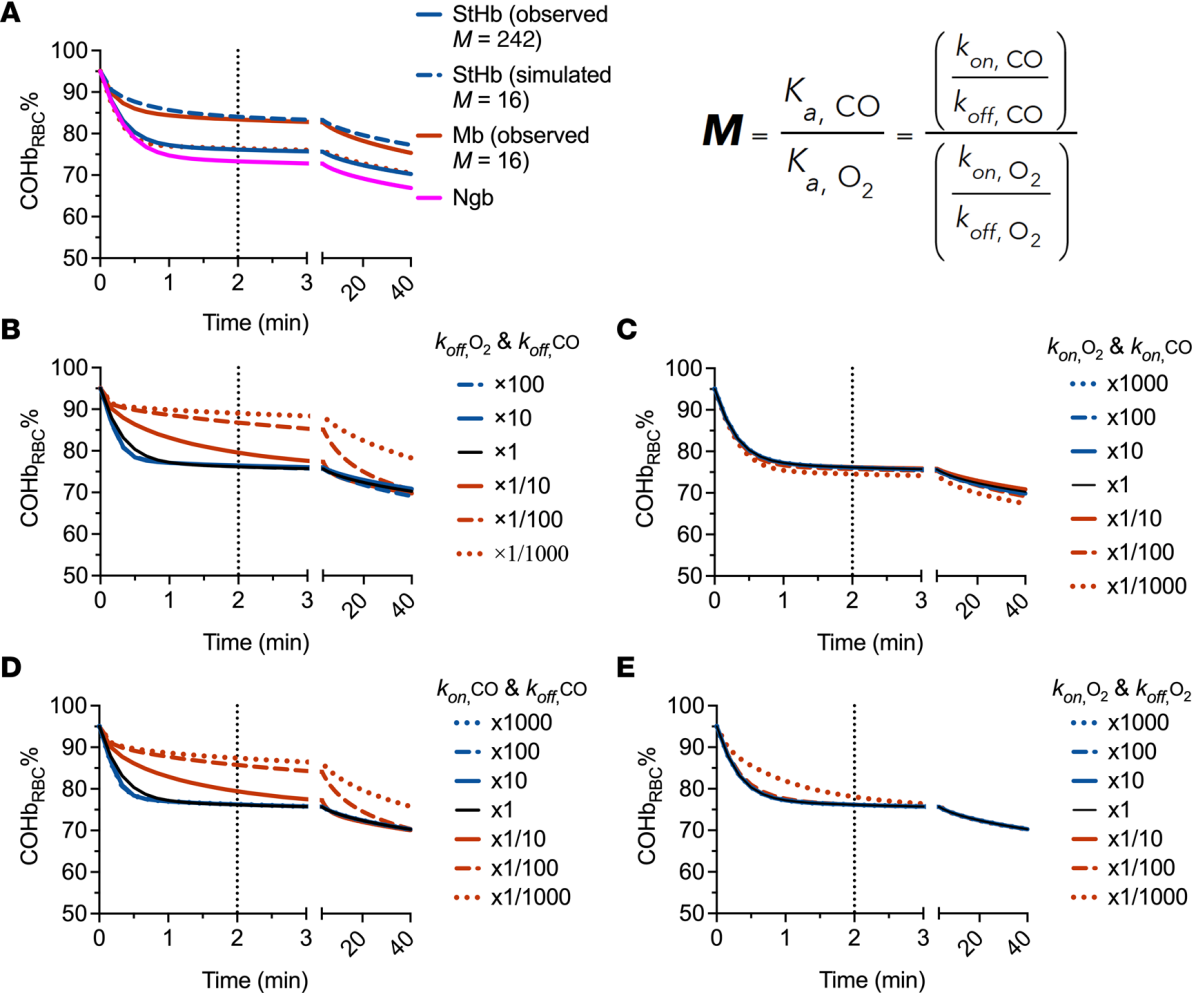
Computationally simulated in vivo scavenging kinetics in lethal–CO-poisoning model. See Supplemental Table 3 for specific parameters used in these models. (**A**) Comparison of initial (2–3 minutes) scavenging kinetics by oxyStHb (solid blue line), oxyMb (solid orange line), and Ngb-H64Q-CCC (solid purple line) in the lethal model, which starts at 95% for COHb_RBC_%. Altering only the *k_off_* of oxygen to adjust the M value (equation), StHb was changed to the M value of Mb (242 → 16, blue hashed lines) and Mb to the M value of StHb (16 → 242, orange hashed lines), resulting in scavenging kinetics approximating one another; however, the kinetic profiles are different, largely due to differences in the other individual rate constants between each protein. **B**–**E** depict scavenging by StHb from CO loaded RBCs (black line, native rate constant values) while increasing (blue lines) or decreasing (orange lines) the individual rate constants of the StHb scavenger in ways to maintain a constant M value at 242. (**B**) Increasing *k*_off_ values concomitantly for CO and oxygen does not change scavenging kinetics much; however, decreasing *k_off_* values slows the scavenging rate. (**C**) If instead, the *k_on_* values are both increased or decreased simultaneously, no change is observed as the limitations stem from release of CO from the RBC and competition for oxygen and CO binding to the scavenger changes analogously. (**D**) Altering the *k_on_* and *k_off_* of CO in a manner where they generate the same affinity (*K_A_*) results in scavenging kinetics similar to **B**. (**E**) Changes in the *k_on_* and *k_off_* of oxygen the same amount does not alter the predicted rate of scavenging.

To determine the important kinetic parameters for design of an ideal in vivo CO scavenger, we modeled a series of theoretical scavengers. Intuitively, a scavenger with high selectivity for CO over oxygen (large M value), will effectively sequester CO under aerobic conditions. This point is illustrated when comparing the in vivo scavenging kinetics of StHb and Mb, simulated using experimentally determined rate constants (Supplemental Table 3 and Figure 8A). StHb has a native M value of 242 and, therefore, sequesters a larger fraction of CO in the first 3 minutes (larger area between the curves with the PBS control) compared with Mb, with an M value of 16. Thus, increases in the M value term are of paramount importance for CO design of a CO scavenger.

In a sensitivity analysis whereby the M value of StHb was readjusted to 16 (hashed blue line) and the M value of Mb readjusted to 242 (dotted orange lines) by adjusting their respective *k*_off,O2_ (Figure 8A) does not result in overlapping curves — i.e., the “StHb with M value of 16” does not overlap the line of Mb and “Mb with M value of 242” does not overlap the line of StHb. This observation suggests that individual scavenger kinetic parameters do influence overall CO scavenging rate and efficacy in the first several minutes after treatment. To elucidate how each kinetic parameter alters efficacy for exogenous CO scavengers, we modeled CO elimination kinetics from RBCs via physiological gas transport and CO_seq_ by simulated scavenger molecules (Figure 8, B–E). We based kinetic parameters for simulated scavengers on those of StHb (native rate constant values), holding M value constant at 242), while increasing or decreasing the individual rate constants simultaneously to maintain that M value. All constants associated with RBC-encapsulated hemoglobin remain unchanged for all simulations. Increasing the values of *k*_off_ concomitantly for CO and O_2_ (Figure 8B) did not significantly alter scavenging kinetics; however, decreasing the values of *k_off_* slowed the scavenging rate. With the former, the scavenging rate increase was limited by the rate of the CO dissociation from hemoglobin in RBCs and subsequent diffusion to extracellular scavenger molecules in circulation. As *k_off_* of oxygen decreased, the scavenger bound more readily available oxygen with higher affinity, moderating the theoretical scavengers’ ability to bind CO. If, instead, the *k_on_* values for both CO and oxygen were increased or decreased simultaneously (maintaining the constant M value of 242), no changes in rate were observed (Figure 8C), likely due to the limitations stemming from CO release from the RBC-encapsulated hemoglobin and no change in competition for oxygen and CO binding. Altering the *k_on_* and *k_off_* of CO in a compensatory manner that maintains the same CO affinity (Figure 8D) yielded scavenging kinetics akin to Figure 8B. Here, oxygen rate constants were unchanged, so CO scavenging was instead dependent upon *k_on,CO_*, and lowering *k_on,CO_* of the scavenger decreased the rate of scavenging under these circumstances. Finally, altering the *k_on_* and *k_off_* of oxygen in a compensatory manner that maintains the same oxygen affinity did not alter the predicted rate of scavenging. As oxygen affinity remained unchanged, the rate of CO scavenging was dependent upon the loss of CO by RBC-encapsulated hemoglobin, as well as binding affinity for CO by the scavenger; these parameters remained unchanged by the changes in these conditions.

## Discussion

In the present work, the CO scavenging efficacy of 3 hemoproteins — StHb, NEMHb, and Mb — was investigated as treatments for CO poisoning. While our previous study demonstrated that a hemoprotein with very high CO affinity and selectivity (Ngb-H64Q-CCC) functions as an effective antidote against CO poisoning (9), here, a more comprehensive study details the CO binding efficacy of 3 readily available hemoproteins with moderate CO binding properties in vitro and in vivo. Tolerability of NEMHb and StHb in toxicity studies demonstrated safety and tolerability of these hemoproteins as CO-poisoning therapeutics. An experimentally validated kinetic model is also presented that predicts in vivo CO binding efficacy based on CO and oxygen affinity binding parameters determined in vitro. This model was utilized to elucidate how differential ligand affinities govern CO scavenging efficacy in blood, providing insight into ideal biophysical properties of CO scavenging systems.

Ligand selectivity for CO over oxygen significantly influenced the extent of CO transfer from RBC-encapsulated hemoglobin to extracellular hemoprotein scavengers. The extent of CO scavenging was greater for StHb, NEMHb, and Mb under anaerobic conditions in vitro compared with aerobic conditions in vitro (Figure 1). This observation reflects relative ligand binding affinities determined from kinetic binding and dissociation rate constants, which also suggests modest selectivity for CO over oxygen in these hemoproteins, especially compared with Ngb-CCC-H64Q (Table 1). Moreover, CO selectivities for StHb (M = 242) and NEMHb (M = 231) are more than an order of magnitude higher than that of Mb (M = 16). As a result, a large discrepancy in CO scavenging under aerobic conditions was observed between StHb and NEMHb (absolute change in COHb_RBC_% = 52.6%) and Mb (absolute change in COHb_RBC_% = 28.7%). Importantly, in vitro total CO scavenging under aerobic conditions accurately reflected in vivo outcomes: CO scavenging efficacy and survival were significantly lower for Mb compared with StHb and NEMHb in vivo in our severe–CO-poisoning mouse model (Figure 2).

Despite exhibiting similar CO affinities and selectivities only slightly greater than those of hemoglobin in RBCs, we observed significant CO binding to both StHb and NEMHb, as well as improved dose-dependent survival outcomes in our severe–CO-poisoning model. Not surprisingly, the highest dose (150 μmol/kg) resulted in the highest survival (100% for StHb and NEMHb) in our severe model (Figures 4, C and D). These survival outcomes are similar to those observed in our previously published Ngb-H64Q-CCC study, where survival at a 120 μmol/kg dose was 87.5% in a similar severe-poisoning model, though formal dose-response studies were not completed using Ngb-H64Q-CCC (9). As both StHb and NEMHb exhibit significantly lower affinity for CO and poorer selectivity for CO over oxygen compared with Ngb-H64Q-CCC (Table 1), similar therapeutic effects in terms of mortality rescue were unexpected. In vitro and in vivo data show that Ngb-H64Q-CCC was the most efficacious scavenger and would require smaller doses for the same amount of CO removal (Figure 7, A and C, and Figure 8A). Our results indicate that other molecules with lower CO affinity and more directly produced as StHb and NEMHb could also provide benefits, although more development is necessary to determine therapeutic window and drug product stability.

Considering that tissue oxygen deprivation is a major downstream effect of CO toxicity (1), a possible explanation for the improved mortality observed in mice treated with StHb and NEMHb may be that these proteins exhibit multiple functionalities of CO_seq_, CO elimination from the cardiopulmonary circulation, and oxygen delivery. Under similar conditions in the severe–CO-poisoning mouse model, heme sites in Ngb-H64Q-CCC are almost fully saturated (99.9% ± 0.1%) by CO (9), while only 65.9% of StHb and NEMHb heme sites are CO bound (Figure 2D). Sites not occupied by CO may participate in oxygen delivery to alleviate tissue hypoxia. The CO-bound StHb and NEMHb may also release CO in the pulmonary circulation, resulting in elimination of CO from the body, and rebind oxygen in the oxygen-rich environment/alveoli after the cessation of CO gas exposure. Cell-free hemoglobin is more efficient at transporting oxygen than RBCs with the same concentration of hemoglobin (21), and previous reports have shown that cell-free hemoglobin at concentrations as low as 0.9 mM (1.5 g/dL, 10.7% of the normal concentration of hemoglobin in blood) delivers sufficient oxygen to sustain normal myocardial function at normal or lower partial pressures of oxygen (22). Thus, StHb and NEMHb may not only remove CO from RBCs, but may also enhance oxygen delivery to tissues, resulting in a beneficial therapeutic rescue response commensurate with that observed in Ngb-H64Q-CCC, which sequesters a larger fraction of CO from RBC in circulation than hemoglobin-based scavengers. Additionally, it is possible that, when these hemoproteins unload bound CO in the pulmonary circulation with exhalation, they may scavenge additional equivalents of CO.

The aforementioned severe–CO-poisoning mouse model has an extremely high COHb_RBC_ level before infusion (9), which is rarely seen in human clinical scenarios, where just 25% COHb levels are severe and associated with acute mortality (5). To better investigate the efficacy of these hemoproteins in COHb_RBC_ levels more akin to human poisoning, therapeutic hemoprotein was administered at lower COHb_RBC_ in mice by (a) reducing CO exposure time and (b) increasing the latency period between CO exposure and infusion. Estimated values for CO_seq_ for NEMHb and StHb do not vary significantly as a function of initial COHb levels in this moderate–CO-poisoning model. Furthermore, CO_seq_ values observed in moderate CO models are comparable with those observed at the same dose of NEMHb and StHb in the severe model, where the initial COHb_RBC_ is ~97% (Figure 3, D and E). These results suggest that the CO binding efficacy of StHb and NEMHb is largely independent of COHb levels and that these hemoprotein scavengers could, therefore, serve as effective therapeutics at COHb_RBC_ levels relevant to humans with severe CO poisoning.

Although the current study focuses on the removal of CO from RBCs, hemoproteins could treat CO poisoning by other mechanisms. Our previous work has proved that Ngb-H64Q-CCC not only enhances clearance of CO from RBCs, but also scavenges CO from cytochrome *c* oxidase and attenuates CO-induced inhibition of mitochondrial respiration (10). Similarly, StHb and NEMHb could also achieve the therapeutic effects by restoring mitochondrial function affected by CO, although future studies are required to test this hypothesis.

Cell-free hemoglobin is well known for toxicity related to circulating hemoglobin monomers and hypertensive effects due to NO scavenging — these risks require further studies and may limit the use of hemoglobin-based CO scavengers. However, bolus infusion in our study resulted in very high initial concentrations at the end of infusion (Figure 5C) and 6 hours after infusion (Supplemental Figure 7). In these conditions, the Hb will be mostly in the tetramer state as the association constants for the tetramer-dimer equilibrium are around 2 × 10^–6^ M (23). In terms of NO scavenging, the presence of high CO levels does decrease the hypertensive effect significantly. MAP traces do not indicate blood pressure increases in the treated mice (Figure 4, A and B) or when considering only surviving mice (Supplemental Figure 3A). We observed a similar phenomenon for Ngb-H64Q-CCC, also attributed to a faster reaction with CO due to the much higher concentration of CO versus NO (9). It is conceivable that some hypertensive effect will be present, as a portion of the injected Hb remains in the oxygen-bound form; however, as noted above, we did not observe hypertensive effects in the MAP traces. We conclude that any hypertensive effect is small in comparison with the hypotensive effect induced by the moderate and severe CO exposures in mice.

Finally, we attempted to computationally model the in vivo effects of potential hemoprotein therapeutic targets for CO poisoning using simple molecular data gathered in vitro. In the case of the exogenous StHb and NEMHb, simplification of the kinetic model for the in vivo study includes the assumption of a constant R-state. The binding parameters for RBC were adopted from the reported value for hemoglobin in R-state. The mathematical prediction for percentage of CO_seq_ by exogenous scavenger and decrease in COHb_RBC_ shows significant accuracy for different hemoproteins in the severe–CO-poisoning model (Figure 7, C and D) and for varied doses of StHb (Figure 7, E and F).

The kinetic model provides a relationship between CO and oxygen binding affinities of a hemoprotein molecule and the therapeutic effects in vivo, allowing exploration of the impact of changes in the M value and individual binding and dissociation parameters. The simulation of varying M values suggests that the M value is strongly correlated with CO scavenging efficacy; the difference in CO scavenging efficacy between StHb and Mb was due to their different M values (Figure 8A). Intuitively, it is possible to improve the efficacy of CO scavenging by increasing the M value, by changing only one of the rate constants for CO and/or oxygen. While the individual rate constants do slightly change CO scavenging efficacy (Figure 8, B–E), any 2 scavengers with similar M values— no matter how the individual rate constants compare — behave similarly in our modeling (within a reasonable range of magnitudes), confirming the importance of differences in M values. Thus, it is possible to use this kinetic model for other hemoproteins with known CO and oxygen affinities to estimate the CO scavenging efficacy in vivo.

There are some limitations in our mathematical model. First, the mathematical approach is based on our specific murine CO-poisoning model instead of human data. Second, the mathematical model does not predict survival or clinical outcome of the CO poisoning — only the CO clearance from RBC encapsulated Hb. Third, although our goal is for the model to be as accurate as possible, we envision this mathematical approach as a first screening to evaluate if a given molecule could be a viable scavenger. Certainly, a predicted idealized scavenger would need to be confirmed experimentally and shown to be both efficacious and safe in multiple preclinical models.

In summary, these studies show that exogenously applied StHb or NEMHb both removed CO from CO-poisoned RBCs effectively in vitro and in vivo, demonstrating an appreciable survival benefit in severely and lethally CO-poisoned mice. The therapeutic effects of StHb and NEMHb in CO poisoning were strongly dose dependent. The observed CO-binding efficacies of StHb and NEMHb were consistent for varying initial COHb_RBC_ levels, including less severe milder models more analogous to the poisoning CO levels observed in humans. Our kinetic model can estimate CO scavenging efficacy in vivo for hemoproteins using measurable intrinsic CO and oxygen affinities. Moreover, preliminary toxicology experiments suggest a favorable safety profile for StHb and NEMHb within the therapeutic window. Taken together, our data indicate that StHb and NEMHb are promising molecules for development of an antidote for CO poisoning and provide insight to the development of future hemoprotein-based therapeutic candidates.

## Methods

Supplemental Methods are available online with this article.

### Preparation of StHb

Expired, leukoreduced human donor packed RBC units were used as a source of hemoglobin. The isolation and purification process was carried out as previously reported (9). Briefly, human RBCs were washed with PBS via 3 rounds of successive centrifugation (at 4,000*g* for 10 minutes at 4°C) and resuspension. The RBCs were then lysed under hypotonic conditions by resuspension in excess deionized water. Cellular debris was removed by centrifugation at 23,000g for 30 minutes at 4°C. The hemoglobin-containing supernatant was passed through a desalting column (Econo-Pac 10DG, Bio-Rad) equilibrated with NS to remove molecules smaller than 6 kDa. The hemoglobin solution was further purified and concentrated by differential ultracentrifugation (Amicon Ultra centrifugal filters, 50 kDa molecular weight cutoff; MilliporeSigma). The isolation and purification processes were conducted at 4°C and completed within 1 week. To assess holoprotein concentration and species fractionation, UV-Visible spectra of freshly prepared hemoglobin samples were deconvoluted in terms standard reference spectra for ferric (Fe^3+^), deoxyferrous (Fe^2+^), and oxyferrous (Fe^2+^-O_2_) hemoglobin. Concentration of hemoproteins reported in this paper refers to heme concentration. UV-Vis spectra were recorded using a Cary 50 spectrophotometer (Varian). Most preparations yielded > 99% Fe^2+^-O_2_ hemoglobin, and any protein batches with more than 1% metHb were discarded. Finally, StHb was filtered using a GD/X 0.2 μm PES filter (Whatman) and stored in aliquots at –80°C.

### Preparation of NEMHb

NEM (Thermo Fisher Scientific, ≥99%) was incubated with hemoglobin, isolated, and purified as described above in a 3:1 ratio of NEM/heme (i.e., in excess of β-Cys93 sites) with gentle rocking (Labnet ProBlot Rocker 25 XLD) for 1 hour at room temperature. Excess NEM was removed by a gravity-flow chromatography column (Econo-Pac 10DG; Bio-Rad) preequilibrated with NS. Then the NEMHb was concentrated by differential ultracentrifugation (Amicon Ultra centrifugal filters, 50 kDa molecular weight cutoff; MilliporeSigma), aliquoted, and stored at –80°C. As above, holoprotein concentration and species fractionation were assessed using UV-Vis spectral deconvolution, and only preparations that yielded > 99% Fe^2+^-O_2_ hemoglobin were stored for further use.

### Direct measurement of the relative affinity constant M values

M values were measured as previously described (19) by the following equation:


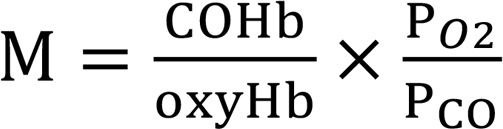


Where COHb and oxyHb were the fraction of COHb and oxyhemoglobin, P_O2_ and P_CO_ were the partial pressure of oxygen and CO in the equilibrium gas phase, respectively. In brief, 2 mL of oxy-StHb or oxy-NEMHb in PBS (pH 7.4) was added to a 4.9 mL quarts cuvette capped with septum. Final concentration of StHb or NEMHb was around 20 μM. CO gas with known concentration in air was injected to the headspace of the cuvette. Equilibrium between gaseous and aqueous phase was reached by gently shaking on a plate rotator at 25°C for 90 minutes until the ratio of COHb/oxyHb was not changed. The absorbance spectra of the Hb solution was measured by spectrophotometer (Cary 100) at 25°C. [COHb], [oxyHb], and [metHb] concentrations were determined by deconvolution.

### Determination of rate constants for CO on-rate (kon,CO) by laser flash photolysis

The *k*_on,CO_ was determined by laser flash photolysis for StHb and NEMHb as previous described (9). Protein samples were saturated with CO in a degassed phosphate buffer containing 10 mM sodium dithionite at room temperature; they then were photolyzed by a laser pulse. CO recombination kinetics were recorded with submicrosecond time resolution. Absorption at 435 nm was fit to 2-phase decay (second-order kinetics) by GraphPad 9.3.1.

### Determination of rate constants for CO dissociation (koff,CO) from StHb and NEMHb

The *k*_off,CO_ was determined by absorption spectroscopy kinetics in NO replacement for StHb and NEMHb in PBS at 25°C. Protein samples 50 μM with sodium dithionite 50 μM were saturated with CO and were then mixed with Proli NONOate 2 mM in 2 mm cuvettes. Absorption spectra were recorded by a Cary 100 spectrophotometer (Varian). The absorption at 419 nm were fit to single exponential decay for the values of *k*_off,CO_.

### Determination of rate constants for oxygen dissociation (koff,O2) from StHb and NEMHb

The *k*_off_,_O2_ was measured in a stopped-flow spectrophotometer using CO replacement for oxygenate StHb and NEMHb in PBS at 25°C. Oxygenated protein 5 or 20 μM was mixed with CO-saturated PBS (CO concentration between 0.8 and 1 mM) in a stopped-flow spectrophotometer. The absorption at 421 nm was fit to single exponential decay for the *k*_off,O2._

### In vitro CO transfer from RBC-encapsulated COHb to extracellular hemoproteins

The CO transfer from RBC-encapsulated COHb to cell-free hemoproteins was monitored as previously reported (1). Mouse blood was collected the same day of the experiment from healthy C57BL/6J mice, centrifuged at 2,000g for 1 minute at room temperature to remove plasma, and then washed with PBS 3 times to isolate the RBCs. CO-saturated PBS buffer was prepared by purging with CO gas (Matheson, CP Grade, 99.5%) on ice. To obtain CO saturated RBCs, RBCs were diluted 5 times in CO saturated buffer with 10 mM dithionite to ensure complete reduction of hemoglobin heme sites and prevent adventitious oxygen binding. Degassed PBS buffer was prepared by purging nitrogen gas (Matheson) for 30 minutes. The CO-RBC mixture was centrifuged at 2,000g for 1 minute at room temperature to remove excessive CO. The RBC pellet was then washed by PBS containing 5 mM dithionite via 2 rounds of successive centrifugation (at 2,000*g* for 2 minutes at 4°C) and resuspension to further remove CO in the buffer. The CO saturated RBCs were resuspended in PBS with dithionite for anaerobic experiments or without dithionite for aerobic experiments. The concentration was adjusted to 0.4 mM in heme, measured by UV-Vis spectroscopy.

For anaerobic experiments, a 1 cm quartz cuvette sealed by a cap with septum was filled with nitrogen to maintain an oxygen-free environment. CO-saturated RBCs were equilibrated to the set temperature in the cuvette and were gently stirred. CO transfer was initiated by mixing the hemoprotein solution (NEMHb, StHb, Mb, or PBS as control) with the CO-saturated RBCs in the cuvette with 10 mM dithionite. The ratio of free hemoprotein to hemoglobin encapsulated within the RBCs was 1:1 (by heme concentration). A 100 μL sample was taken out from the cuvette at predefined time points. The sample was then centrifuged at 2,000*g* for 10 seconds at room temperature. The supernatant and the RBC pellet were separated and stored on ice. When all the samples were collected, absorbance spectroscopy was used to measure the saved supernatant and the RBC pellet heme ligand binding state (i.e., oxygen-bound, CO-bound, ferric). The supernatants were measured after diluting with PBS with 10 mM dithionite. The RBC pellets were measured after lysis with 0.5% NP40 in PBS with 10 mM dithionite.

For aerobic experiments, the procedures were the same as the anaerobic experiments, except that no dithionite was used in the making and washing of CO-saturated RBCs and reaction mixtures, and the cuvette was not capped or purged with nitrogen; the reaction system was open to air, and oxygen partial pressure in the reaction mixture was estimated to be 160 mmHg.

UV-Vis spectroscopy was used to determine the transfer of CO between RBC-encapsulated COHb and extracellular hemoproteins. Concentration and species fractionation were assessed using UV-Vis spectral deconvolution with the CO saturated RBCs lysed by hypotonic 20 mM sodium dithionite solution. Multiple replicate measurements were performed to ensure the accuracy. Equimolar amounts of fully reduced Mb, NEMHb, or StHb were mixed with mouse RBCs saturated by CO.

### CO-poisoning mouse models

Adult male C57BL/6J mice (age 13.5 ± 0.2 weeks, body weight 28.9 ± 0.2g; The Jackson Laboratory) were used for all animal experiments. Mouse blood was collected and used in the same day for the in vitro CO-RBC experiments.

In vivo therapeutic effects of hemoproteins in moderate and severe CO poisoning were investigated, adapting previously described mouse models (9). Prior to and after exposure, mice were ventilated with room air (21% oxygen). Premixed 3% CO gas used for exposure experiments was obtained from Matheson Inc., and it contained 3.0% (30,000 ppm) CO, 21% oxygen, and 76% nitrogen. The CO gas mixture was inhaled through the ventilator for a preset duration for different models.

One severe–CO-poisoning model and 2 moderate–CO-poisoning models were tested in this project. For the severe model (Figure 2A), the CO inhalation lasts 4.5 minutes, followed by a 2-minute venous infusion of hemoprotein, NS, or PBS. For the 2 moderate models (Figure 5A), the CO inhalation lasts 1.5 minutes. In Moderate model A, the 2-minute infusion starts at 4.5 minutes, which is 3 minutes after the CO exposure. In Moderate model B, the infusion starts at 24.5 minutes, which is 23 minutes after the CO exposure.

For in-life studies, mice were anesthetized with 2%–3% isoflurane. An incision was made on the neck. A tracheal tube was introduced via a tracheotomy for mechanical ventilation with a 20 gauge cannula. Catheters (0.025” × 0.012”) were inserted into the right carotid artery and jugular vein. The incision was closed with 6-0 sutures. The anesthetized mice were then moved into a fume hood to protect the operators from CO poisoning. Volume-controlled mechanical ventilation (fraction of inspired oxygen 21%, tidal volume 8.8 mL/kg body weight, 175 breaths per minute) was applied with a small animal ventilator (MiniVent, Type 845; Hugo Sachs). Arterial blood pressure was continuously recorded (PowerLab 8/35, ADInstrument) via the arterial catheter. The MAP and pulse rate were calculated by Labchart software (ADInstrument).

Arterial blood samples were taken through the arterial catheters at preset time points to determine COHb levels and plasma protein levels. A total of 15 μL of blood was taken each time with a Hamilton syringe (Hamilton Company). Blood was centrifuged for 1 minute at 10,000g at room temperature to separate the plasma from cells. Then, the plasma and cell pellet were frozen on dry ice and store in –80°C for later analysis.

Venous injection was controlled by an injection pump with the volume of 10 mL/kg body weight and the injection time of 2 minutes for both test articles (NEMHb, StHb, Mb) and control (NS or PBS). NS was used as control group for the severe–CO-poisoning model, including the hemoprotein comparison study and the dose-response study of StHb and NEMHb. PBS was used in the moderate–CO-poisoning models. Hemoproteins were administered in the oxygenated ferrous form in the treatment groups with a concentration of 10 mM heme, to achieve a dose of 100 μmol/kg body weight, except for the dose-response study of StHb and NEMHb.

### Quantification of hemoprotein concentrations and species distribution

Mouse blood samples (15 μL) withdrawn in the CO-poisoning study were immediately centrifuged at 10,000*g* for 1 minute to separate the RBCs pellet and plasma. Samples were then immediately frozen and stored at –80°C. To determine the fraction of CO-bound hemoglobin, each frozen blood cell pellet was thawed and lysed by dilution into a hypotonic 20 mM sodium dithionite solution to prevent adventitious oxygen binding and facilitate spectral deconvolution. To determine the fraction of CO-bound extracellular scavenger, frozen plasma was thawed and diluted into 20 mM sodium dithionite solution to facilitate spectral deconvolution. Absorption spectra of lysed RBC and diluted plasma samples were recorded from 450–700 nm using a Cay 50 spectrophotometer (Varian). Spectral deconvolution was employed to determine the concentrations and relative fractions of hemoprotein species for RBC-derived hemoglobin and extracellular hemoproteins using standard references for Fe^3+^, Fe^2+^, Fe^2+^-O_2_, and CO-bound species, along with a linear scattering correction (10).

### Blood concentration calculation and estimation

Blood concentration of RBC-encapsulated hemoglobin and plasma concentration of exogenous hemoprotein were measured directly with RBC lysates or plasma, respectively, and calculated by dilution factors. Blood concentration of exogenous hemoprotein were then calculated from measured plasma concentration and plasma volume percent of the whole blood, which was 100% minus hematocrit (e.g., [Blood CO-bound scavenger] = [Plasma CO-bound scavenger] × [100% – hematocrit]). Considering the correction for blood dilution by infusion, estimation of CO_seq_ is as follows.


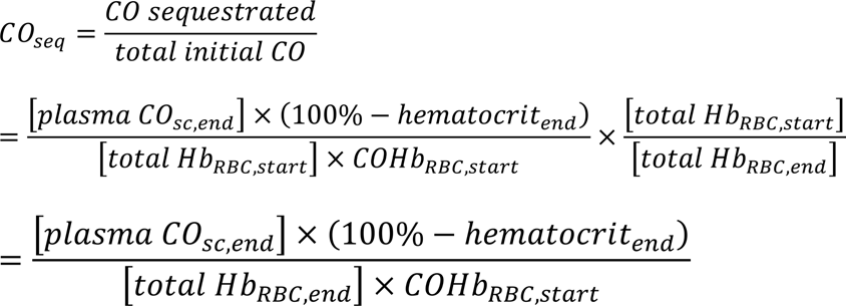


Where [Blood CO-bound scavenger] and [plasma CO-bound scavenger] are the blood concentrations and plasma concentration of CO-bound scavenger at the end of infusion, respectively. [plasma CO_sc,end_] is the plasma concentration of CO-bound hemoprotein scavenger at the end of infusion. Hematocrit_end_ is the hematocrit at the end of infusion, [total Hb_RBC,start_] and [total Hb_RBC,end_] is the blood concentration of total RBC-encapsulated hemoglobin at the start and end of infusion, and COHb_RBC,start_ is the RBC-encapsulated COHb percent at the start of infusion.

Absolute change in COHb_RBC_% (after infusion) = COHb_RBC_ % (start infusion) – COHb_RBC_% (after infusion)

### Mathematical model for CO transfer from RBCs to cell-free hemoproteins

#### Mathematical model.

CO transfer from RBC-encapsulated COHb to extracellular hemoproteins was simplified to a single- compartment system containing RBCs and extracellular hemoproteins. Reactions were described in Equations 1–5.

#### Parameter determination.

Equations 1–5 were used to generate a kinetic model using the COPASI software, version 4.29 (20). Total volume of the system was set to be 2.0 mL for in vitro studies and 2.5 mL for in vivo studies based on the estimated whole blood volume for a C57BL/6J mouse by 79 mL/kg body weight (24). Oxygen concentration was set to be 0.21 mM according to the normal arterial blood oxygen partial pressure (25).

Kinetic rate constants for ligand binding (*k*_on_) and ligand dissociation (*k*_off_) for RBC-encapsulated COHb and exogenous CO scavengers were determined from in vitro experiments and used to compute ligand affinity values (Table 1). The model assumes that all hemoglobin proteins (RBC-encapsulated COHb and exogenous StHb and NEMHb) are in the R-state; therefore, R-state affinity parameters were utilized. For the mouse RBCs, rate constants were assumed to the reported value of hemoglobin R-state.

Binding and dissociation rate constants, initial protein, and ligand concentrations are summarized in Supplemental Table 3.

The only unknown parameter, *k*_COloss_, was determined from changes in COHb_RBC_ of PBS control experiments in vitro and NS control groups from the hemoprotein comparison and StHb dose-response studies in vivo. Using the built-in parameter estimation functions of COPASI, the RBC-encapsulated COHb and CO binding values were fitted to find the best *k*_COloss_ using the Genetic Algorithm and Evolutionary Programming methods. The *k*_COloss_ was calculated to be 0.01259 s^–1^ for in vitro study and 6.67 s^–1^ for in vivo study.

#### Validation.

The validation sets are data of the hemoprotein comparison study, StHb dose-response study, and the moderate–CO-poisoning study. These data sets exam the performance of the model in situations of different hemoproteins, different doses, and different initial RBC-encapsulated COHb levels. Substituting the observed data of blood concentration of extracellular hemoprotein, initial RBC-encapsulated COHb%, and RBC hemoglobin concentration, the CO binding and RBC-encapsulated COHb percentage were predicted. Predictive performance of the model was assessed by the coefficient of determination *R*^2^, slope, and Y-intercept of regression, and RMSE.

### Toxicological study

Healthy C57BL/6J mice were anesthetized with inhaled isoflurane and body temperature maintained at 37°C. A catheter was implanted into the lateral tail vein, through which the Fe^2+^-O_2_ NEMHb and StHb preparation solution was infused in 30 minutes by an injection pump. The catheter was removed, followed by ligation of the vessel and closure of the incision. Mice were then placed in cages and observed for 48 hours, monitoring activity, nesting, and daily weights. The mice were euthanized after 48 hours. At necropsy, blood samples were sent for complete blood count (MASCOT Hemavet 950 Veterinary hematology Analyzer) and serum chemistry measurement (Marshfield Labs).

### Statistics

Data are shown in the form of mean ± SEM and analyzed with GraphPad Prism software version 8.4.2. Unpaired 2-tailed *t* test was used for comparison of 2 groups and normal distribution. One-way or 2-way ANOVA was used for comparison of more than 2 groups. Kaplan-Meier survival curves were compared with log-rank (Mantel-Cox) test. Bonferroni corrected threshold for *P* value was adopted for each individual comparison in the survival curves. MAP data were compared by 2-way repeated-measures ANOVA. Agreement between the prediction and observation was analyzed by Spearman correlation. * shows *p* value of multiple comparison of Tukey test (**** *P* < 0.0001, *** *P* < 0.005, ** *P* < 0.01, * *P* < 0.05). A *p* value less than 0.05 was considered significant.

### Study approval

All animal studies were performed in accordance with protocols approved by the IACUC at the University of Pittsburgh and NIH Guide for Care and Use of Laboratory Animals.

## Author contributions

MTG conceived and supervised the project. QX designed and performed all in vitro experiments and some in vivo experiments. JJR designed and performed some in vivo experiments and assisted with critical review of the manuscript. XC performed most in vivo experiments. LW performed some in vivo experiments. AWD assisted with the in vitro experiments and development of the kinetic model. MRD assisted with the mathematical simulation. ST isolated, purified, and made the StHb and NEMHb. KB assisted with the in vivo experiments. XNH assisted with some in vitro experiments. QT assisted with some in vitro experiments. CFM assisted with experimental design. LG assisted with in vivo experiments. EA, TCJ, and KBU conducted laser photolysis and NO displacement experiments and analyzed data. DBKS designed and supervised laser photolysis and NO displacement experiments. JT conducted mathematical simulation and critical review of the manuscript.

## Supplementary Material

Supplemental data

## Figures and Tables

**Table 1:**
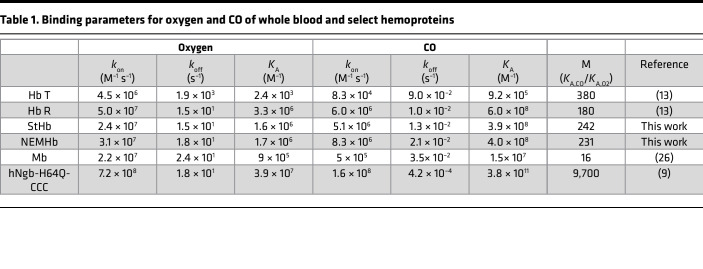
Binding parameters for oxygen and CO of whole blood and select hemoproteins

## Version 1 09/29/2022

In-Press Preview

## Version 2 11/08/2022

Electronic publication
