# Supplementary material for: Cell-free and alkylated hemoproteins improve survival in mouse models of carbon monoxide poisoning
Source: JCI Insight. 2022 Nov 8;7(21):e153296. doi: 10.1172/jci.insight.153296 (PMC9675481; doi:10.1172/jci.insight.153296)
Supplement: Supplemental data [file jciinsight-7-153296-s298.pdf]

Supplemental methods

Preparation of Mb

Isolated equine Mb was obtained commercially as a lyophilized powder (Sigma-Aldrich). Solid protein was dissolved in PBS and centrifuged at 18,000 ×g to remove any undissolved protein. Excess dithionite (ACROS, 85%, tech) was added to the ferric metMb to reduce the heme and then removed by passing the solution through desalting column (Econo-Pac 10DG, BioRad) preequilibrated with NS. The reduced Mb quickly bound dissolved oxygen in the preequilibrated column buffer, and Mb heme in the column effluent was almost entirely (>99%) oxygen-bound, as determined by UV-Vis spectroscopy. The resulting oxyMb solution was concentrated by differential ultracentrifugation (Amicon Ultra centrifugal filters, 10 kDa molecular weight cutoff, Millipore) at 4 °C. Aliquots were stored at -80 °C until further use.

Hematocrit measurement:

Arterial blood was collected into a 1.5 mL tube (Safe-Lock Tubes, Eppendorf). Heparinized capillary tubes (Micro-Hematocrit Cappillary tubes, Fisherbrand) were filled with the blood by capillary forces. One end of the capillary tube was sealed thoroughly by vinyl plastic putty (Leica CRITOSEAL). Total length of the sample and the length of the packed RBCs were measured of the capillary tube after three minutes of centrifugation (Jouan Microcentrifuge A13). Hematocrit was calculated by the length ratio of the packed RBCs to the total sample in the capillary tube.

Time course of NEMHb clearance

Pharmacokinetic data was collected in a 72-hour study. NEMHb solution was administered by IV with the dose of 100 μmol/kg (preparation in 4 mM, 25 ml/kg). The IV duration was 30 minutes. The same volume of NS without hemoproteins was used as control group. 15 μL of blood was

collected by the dwelling catheter in the femoral artery at preset timepoints. Plasma concentration of NEMHb was measured by UV-Vis spectroscopy. Blood concentration was then calculated by plasma concentration × (1- hematocrit) = plasma concentration × 0.55.

Supplemental Table 1. Summary of scavenger-dependent changes in $COHb_{RBC}$ in severe CO poisoning mouse model.

Scavenger	$COHb_{RBC, Start}$ (%)	$COHb_{RBC}$ (%)	$\|\Delta COHb_{RBC}\|$ (%)	[total scavenger] (mM)	[total RBC hemoglobin] (mM)	CO_{seq} (%)
NEMHb	95.8 ± 0.2	79.4 ± 1.0	16.4 ± 1.0	1.1 ± 0.1	6.3 ± 0.3	11.5 ± 1.3
StHb	96.1 ± 0.4	79.0 ± 0.8	17.1 ± 0.9	1.0 ± 0.1	6.2 ± 0.2	10.9 ± 0.8
Mb	96.2 ± 0.5	85.9 ± 0.4	10.3 ± 0.3	0.8 ± 0.1	5.8 ± 0.3	2.5 ± 0.5
NS	91.5 ± 1.3	85.6 ± 1.2	5.9 ± 1.9	0.0 ± 0.0	5.8 ± 0.3	0.0 ± 0.0

Quantities are value at the end of infusion unless indicated otherwise. $COHb_{RBC,start}$ shows percent of carboxyhemoglobin in RBCs at the start of infusion. Concentrations show blood concentration.

Supplemental Table 2. Behavior, body weight and complete blood count in the 48-hour safety study

		NS	StHb	NEMHb	Normal range	Unit	*p* value
Behavior	0	normal	normal	normal			
	24hr	normal	normal	normal			
	48hr	normal	normal	normal			
Body Weight	0	29.4 ± 1.0	27.5 ± 0.8	29.5 ± 0.7		g	
	24hr	29 ± 0.9	26.6 ± 0.9	28.8 ± 0.6		g	0.1232
	48hr	29.2 ± 1.0	26.9 ± 0.8	29.4 ± 0.5		g	
Complete	WBC	5.5 ± 0.9	3.5 ± 0.4	3.8 ± 0.5	1.8 - 10.7	K/µl	0.1168
Blood Count	RBC	8.7 ± 0.2	8.7 ± 0.5	8.7 ± 0.3	6.4 - 9.4	M/µl	0.9976
	Hb	12.5 ± 0	11.96 ± 0.7	12.5 ± 0	11 - 15.1	g/dL	0.6052
	Hematocrit	42.0 ± 1.8	38.5 ± 2.6	42.8 ± 1.3	35.1 - 45.4	%	0.2858
	Platelet	341.2 ± 42.3	324.4 ± 110.3	465.6 ± 26.13	592 - 2972	K/µl	0.3296

Data are presented as mean ± SEM for N=5 in each group. *P* values were determined using one-way ANOVA for each row except the body weight, which was by two-way ANOVA.

Supplemental Table 3. Parameters of the kinetics model

model	CO scavenger (X)	$k_{on,CO,R}$ ($M^{-1}\,s^{-1}$)	$k_{off,\,CO,\,R}$ (s^{-1})	$k_{on,O2,R}$ ($M^{-1}\,s^{-1}$)	$k_{off,\,O2,\,R}$ (s^{-1})	$k_{on,CO,X}$ ($M^{-1}\,s^{-1}$)	$k_{off,\,CO,\,X}$ (s^{-1})	$k_{on,O2,X}$ ($M^{-1}\,s^{-1}$)	$k_{off,\,O2,\,X}$ (s^{-1})	[R] (mM)	$COHb_{RBC}$ (%)	[XO2] (mM)
in vitro	StHb	6.0×10^{6}	1.0×10^{-2}	5.0×10^{7}	1.5×10^{1}	5.1×10^{6}	1.3×10^{-2}	2.4×10^{7}	1.5×10^{1}	0.2	96.7 ± 0.6	0.2
	NEMHb	6.0×10^{6}	1.0×10^{-2}	5.0×10^{7}	1.5×10^{1}	8.3×10^{6}	2.1×10^{-2}	3.1×10^{7}	1.8×10^{1}	0.2	95.9 ± 0.4	0.2
	Mb	6.0×10^{6}	1.0×10^{-2}	5.0×10^{7}	1.5×10^{1}	5.0×10^{5}	3.5×10^{-2}	2.2×10^{7}	2.4×10^{1}	0.2	97.4 ± 0.2	0.2
in vivo	StHb	6.0×10^{6}	1.0×10^{-2}	5.0×10^{7}	1.5×10^{1}	5.1×10^{6}	1.3×10^{-2}	2.4×10^{7}	1.5×10^{1}	6.2 ± 0.2	96.1 ± 0.4	1.0 ± 0.1
	NEMHb	6.0×10^{6}	1.0×10^{-2}	5.0×10^{7}	1.5×10^{1}	8.3×10^{6}	2.1×10^{-2}	3.1×10^{7}	1.8×10^{1}	6.3 ± 0.3	95.8 ± 0.2	1.1 ± 0.1
	Mb	6.0×10^{6}	1.0×10^{-2}	5.0×10^{7}	1.5×10^{1}	5.0×10^{5}	3.5×10^{-2}	2.2×10^{7}	2.4×10^{1}	5.9 ± 0.3	96.2 ± 0.5	0.8 ± 0.1

[R] is the blood concentration of total hemoglobin in RBCs, [XO2] is the blood concentration of extracellular or exogenous oxyferrous hemoprotein. [R], $COHb_{RBC}$ and [XO2] are initial values at the start of mixing in in vitro or infusion in in vivo studies, expressed as mean ± SEM. The initial concentration of free CO is assumed to be zero, and that of oxygen assumed to be 0.23 mM.

Supplemental table 4 Predictive performance of the kinetics model.

Data set		n	Slope	Intercept	R^2	RMSE	SD
Hemoprotein comparison study	CO_{seq}	15	0.90 [0.82 - 0.99]	-1.18 [-2.15 - -0.21]	0.98	0.68	5.0
	$\|\Delta COHb_{RBC}\|$		0.85 [0.58 - 1.11]	3.82 [0.17 – 7.5]	0.80	1.50	3.6
StHb Dose-response study	CO_{seq}	30	0.97 [0.93 – 1.01]	-0.45 [-0.99 - 0.07]	0.99	0.76	6.4
	$\|\Delta COHb_{RBC}\|$		0.72 [0.59 - 0.84]	-3.2[0.25 - 4.39]	0.86	2.1	4.4

R^2, coefficient of determination; RMSE, root mean square error; SD, standard deviation of the experimentally measured data; Values of slope and intercept are presented as mean [95% confidence interval].

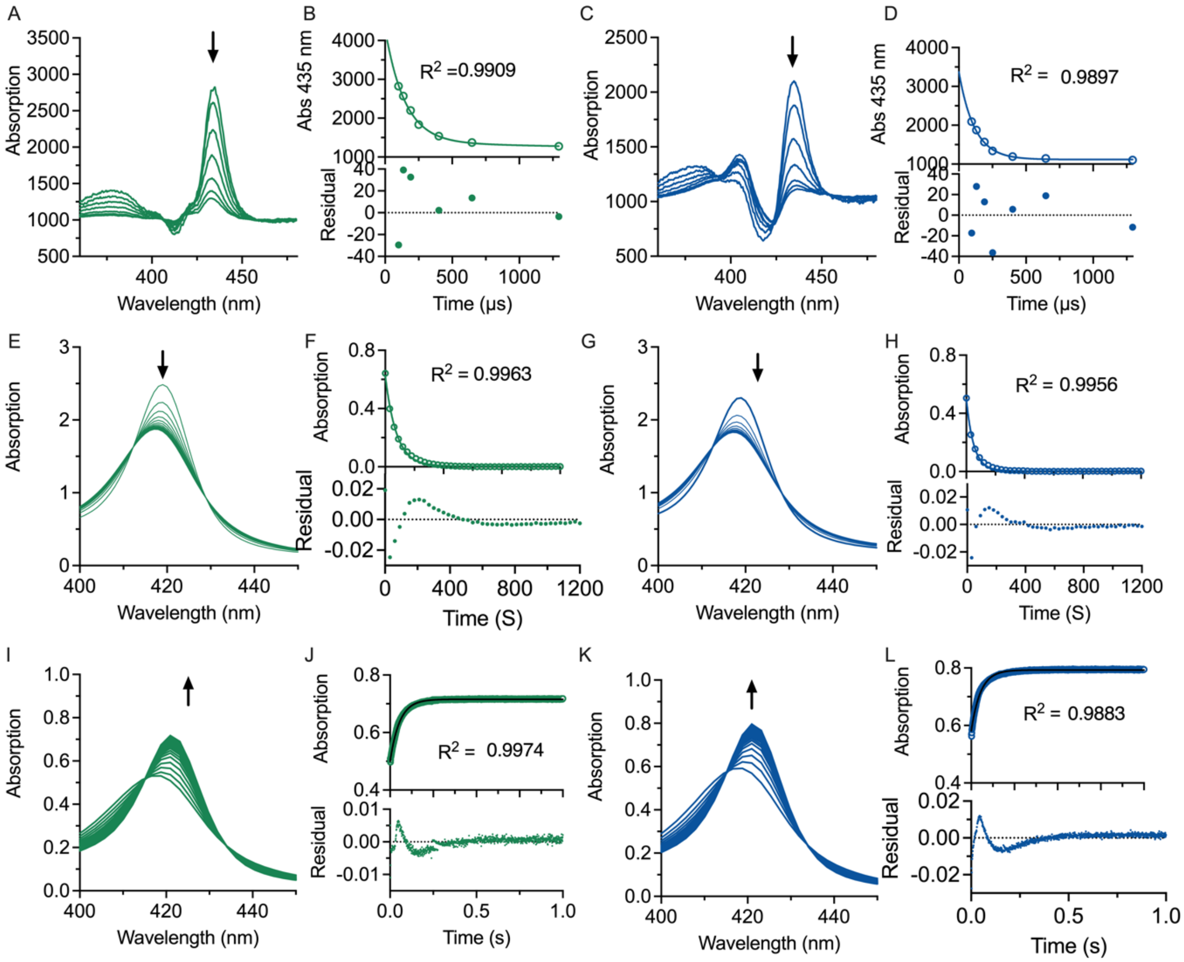

Supplemental Figure 1. Determination of CO binding and dissociation rate constants for StHb and NEMHb. **(A-D)** Rate constants of CO association ($k_{on,\,CO}$) to StHb and NEMHb were measured by laser photolysis. **(A)** Examples of differential spectrum for StHb. (**B**) Top: Absorption at 435 nm of StHb was fit to two phase decay. Bottom: Residuals of the fitting. **(C)** Examples of differential spectrum for NEMHb. (**D**) Absorption at 435 nm of NEMHb was fit to two phase decay. Bottom: Residuals of the fitting. (**E-H**) Measurement of rate constants of CO dissociation ($k_{off,\,CO}$) by NO displacement of carboxy StHb and NEMHb. **(E)** Example of absorption change of carboxy StHb. **(F)** Top: Absorption at 419 nm of StHb was fit to one phase decay. Bottom: Residuals of the fitting. **(G)** Example of absorption change of carboxy NEMHb. **(H)** Top: Absorption at 419 nm of NEMHb was fit to one phase decay. Bottom: Residuals of the fitting. (**I-L**) Measurement of rate constants of oxygen dissociation ($k_{off,\,O2}$) by CO displacement of oxyhemoglobin of StHb and NEMHb. **(I)** Examples of absorption change of StHb. **(J)** Top: Absorption at 421 nm of StHb was fit to one phase decay. Bottom: Residuals of the fitting. **(K)** Examples of absorption change of NEMHb. **(L)** Top: Absorption at 421 nm of StHb was fit to one phase decay. Bottom: Residuals of the fitting.

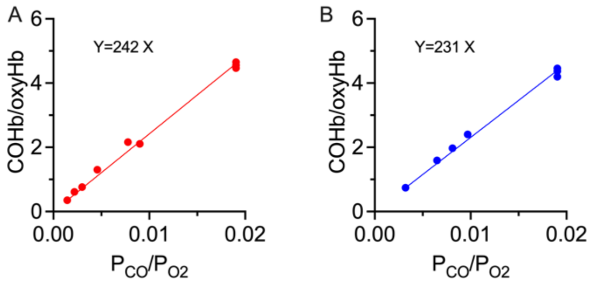

Supplemental Figure 2. Determination of M values by the relationship of COHb/oxyHb to P_{CO}/P_{O2} in the gas phase. Measurement is compared with the solid line representing M value of 242 ± 4 for **(A)** StHb and M value of 231 ± 3 for (**B**) NEMHb.

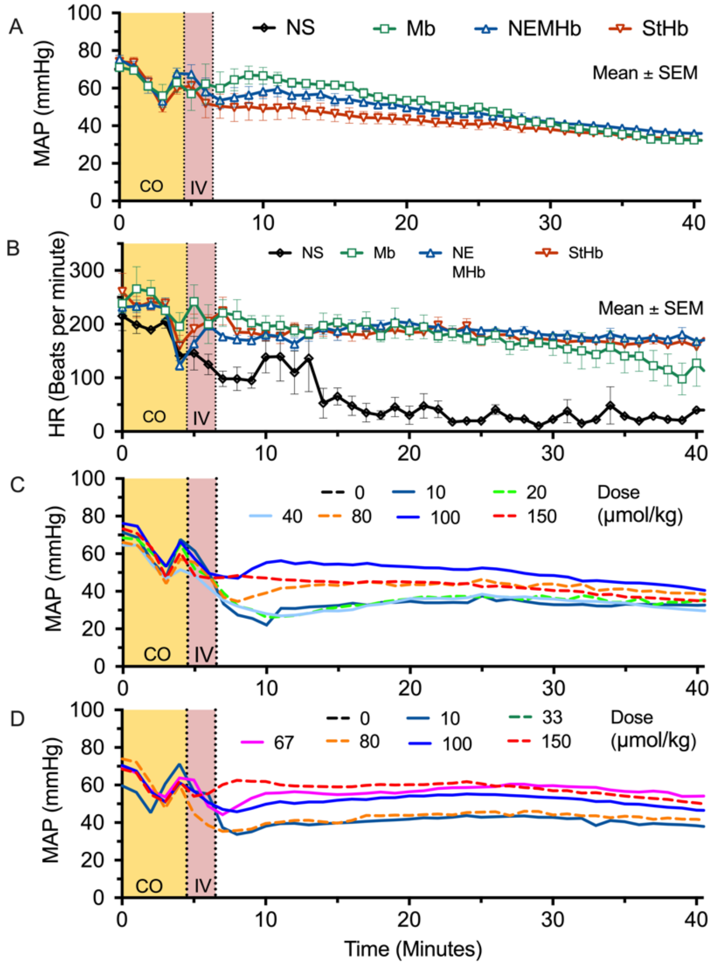

Supplemental Figure 3. Hemodynamic observation of the severe CO poisoning model. (A) Survivor-only MAP of the hemoprotein comparison study in the severe CO poisoning model. (**B**) HR of the hemoprotein comparison study in the severe CO poisoning model. (**C**) Survivor-only MAP of StHb dose-response study (N=5 for each group). (**D**) Survivor-only MAP of NEMHb dose-response study (n=6-9 for each group). "CO" (yellow) indicates the period of 4.5-minute CO inhalation, "IV" (red) indicates the period of 2-minute infusion. Data is shown as mean for each group.

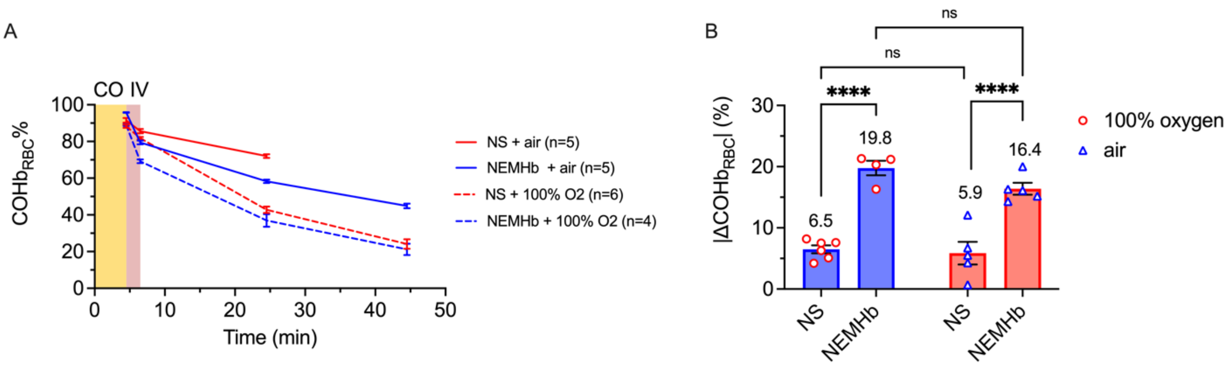

Supplemental Figure 4. Comparison of efficacy of NEMHb with and without supplemental 100% oxygen in severe CO poisoning mouse model. (A) Time course of the $COHb_{RBC}$ % change. 100% oxygen or air is supplemented immediately after 4.5-minute CO inhalation. (**B**) Comparison of $|\Delta COHb_{RBC}|$% between NS and NEMHb with and without supplemental 100% oxygen. There is no significant difference for NEMHb with or without supplemental 100% oxygen by two-way ANOVA (p = 0.2779).

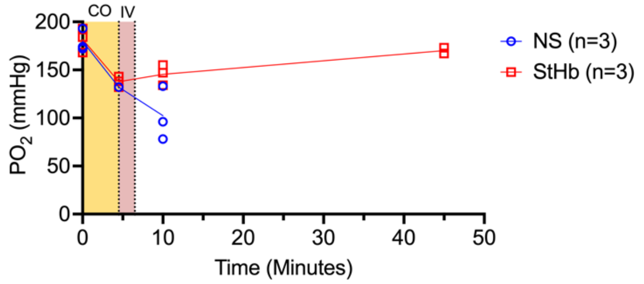

Supplemental Figure 5. Partial pressure of oxygen (PO_2) in the severe CO poisoning model. A recovery of the PO_2 values was observed to almost initial levels by 40 min after StHb treatment. The PO_2 for the animals treated with normal saline kept decreasing after infusion. No surviving animals were present in the NS group for a 45 min test.

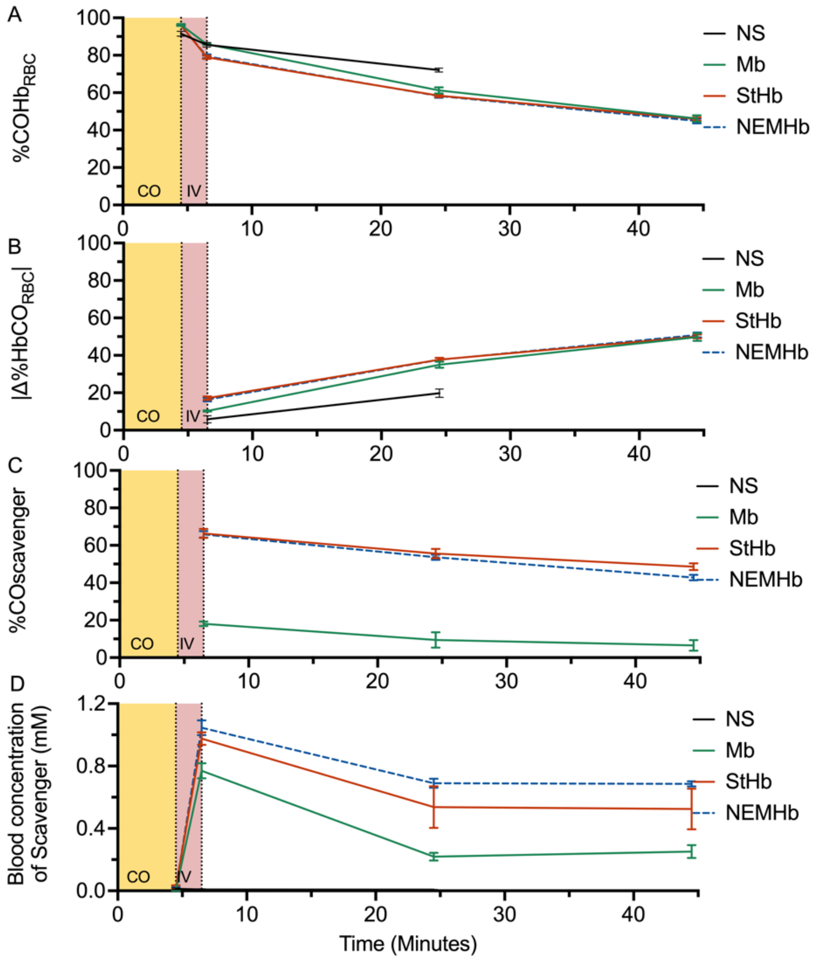

Supplemental Figure 6. Comparison of hemoproteins in a severe CO poisoning mouse model. Time course of the $COHb_{RBC}$**(A)**, $|\Delta COHb_{RBC}|$(**B**), CO_{sc} (**C**), and blood concentration of CO scavengers (**D**). "CO" (yellow) indicates the period of 4.5-minute CO inhalation, "IV" (red) indicates the period of 2-minute infusion. Data is shown as mean ± SEM for N=5 in each group.

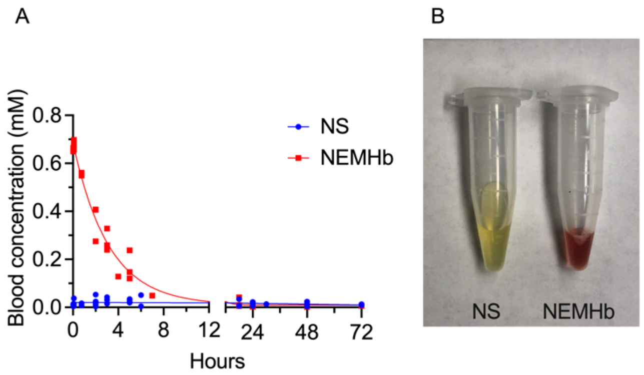

Supplemental Figure 7. 72-hour pharmacokinetics study of NEMHb on healthy mice. (A) NEMHb was administered by IV with the dose of 100 µmol/kg (preparation in 4 mM, 25 ml/kg) to healthy mice not exposed to CO poisoning. The IV duration was 30 minutes. The same volume of NS without hemoproteins was used as control group. Half-life of NEMHb in the plasma was 2.2 hours. N= 11 and 7 for NS and NEMHb, respectively. (**B**) Urine samples of NS and NEMHb. right micro-tube of the picture below, the urine shows significant red color collected 30 minutes after NEMHb injected to a mouse at the dose of 100 µmol/kg. The left tube is the urine collected from a mouse injected with same volume of normal saline.
